# Therapeutic Potential of Stem Cell-Derived Extracellular Vesicles on Atherosclerosis-Induced Vascular Dysfunction and Its Key Molecular Players

**DOI:** 10.3389/fcell.2022.817180

**Published:** 2022-03-18

**Authors:** Ioana Karla Comariţa, Alexandra Vîlcu, Alina Constantin, Anastasia Procopciuc, Florentina Safciuc, Nicoleta Alexandru, Emanuel Dragan, Miruna Nemecz, Alexandru Filippi, Leona Chiţoiu, Mihaela Gherghiceanu, Adriana Georgescu

**Affiliations:** ^1^ Institute of Cellular Biology and Pathology ‘Nicolae Simionescu’ of Romanian Academy, Bucharest, Romania; ^2^ ‘Victor Babeș’ National Institute of Pathology, Bucharest, Romania; ^3^ ‘Carol Davila’ University of Medicine and Pharmacy, Bucharest, Romania

**Keywords:** vascular dysfunction, inflammation, atherosclerosis, cardiovascular diseases, extracellular vesicles, siRNA Smad2/3

## Abstract

Atherosclerosis is a progressive, chronic inflammatory disease of the large arteries caused by the constant accumulation of cholesterol, followed by endothelial dysfunction and vascular inflammation. We hypothesized that delivery of extracellular vesicles (EVs), recognized for their potential as therapeutic targets and tools, could restore vascular function in atherosclerosis. We explored by comparison the potential beneficial effects of EVs from subcutaneous adipose tissue stem cells (EVs (ADSCs)) or bone marrow mesenchymal stem cells (EVs (MSCs)) on the consequences of atherogenic diet on vascular health. Also, the influences of siRNA-targeting Smad2/3 (Smad2/3siRNA) on endothelial dysfunction and its key molecular players were analyzed. For this study, an animal model of atherosclerosis (HH) was transplanted with EVs (ADSCs) or EVs (MSCs) transfected or not with Smad2/3siRNA. For controls, healthy or HH animals were used. The results indicated that by comparison with the HH group, the treatment with EVs(ADSCs) or EVs(MSCs) alone or in combination with Smad2/3siRNA of HH animals induced a significant decrease in the main plasma parameters and a noticeable improvement in the structure and function of the thoracic aorta and carotid artery along with a decrease in the selected molecular and cellular targets mediating their changes in atherosclerosis: **1)** a decrease in expression of structural and inflammatory markers COL1A1, α-SMA, Cx43, VCAM-1, and MMP-2; **2)** a slight infiltration of total/M1 macrophages and T-cells; **3)** a reduced level of cytosolic ROS production; **4)** a significant diminution in plasma concentrations of TGF-β1 and Ang II proteins; **5)** significant structural and functional improvements (thinning of the arterial wall, increase of the inner diameter, enhanced distensibility, diminished VTI and Vel, and augmented contractile and relaxation responses); **6)** a reduced protein expression profile of Smad2/3, ATF-2, and NF-kBp50/p65 and a significant decrease in the expression levels of miR-21, miR-29a, miR-192, miR-200b, miR-210, and miR-146a. We can conclude that **1)** stem cell-derived EV therapies, especially the EVs (ADSCs) led to regression of structural and functional changes in the vascular wall and of key orchestrator expression in the atherosclerosis-induced endothelial dysfunction; **2)** transfection of EVs with Smad2/3siRNA amplified the ability of EVs(ADSCs) or EVs(MSCs) to regress the inflammation-mediated atherosclerotic process.

## Introduction

Atherosclerosis is a chronic, fibroproliferative inflammatory disease that affects the structure and function of large and medium arteries. To this day, it is considered the main cause of cardiovascular disease (CVD), with a very high mortality and morbidity rate in developed countries.

Atherogenesis is a slow, multistage process involving successive events and is characterized by the accumulation of lipids, especially cholesterol crystals in the vascular intima, macrophage infiltration, smooth muscle cell (SMC) proliferation with changing phenotype, accumulation of connective tissue components that by fibroblasts and calcification determine stiffening of the arteries, obstruction of blood flow, and formation of thrombus (Manduteanu & Simionescu, 2012).

Atherosclerosis is a progressive, complex vascular disease with an autoimmune component, which is not only a degenerative consequence, inevitable aging, but also an inflammatory condition, which can be transformed into an acute clinical event, by rupture of the atheroma plaque, clot formation, and by installing thrombosis (Amaya-Amaya, Montoya-Sanchez, & Rojas-Villarraga, 2014). In the context of atherosclerotic disease, there is a tight link between immunological and metabolic processes, especially the interaction between lipids (mainly cholesterol), the way in which activates the cells of the immune system and the associated inflammatory response. The evolution of atherosclerotic disease is induced by the innate and adaptive immune system responses that are regulated by a diversity of cytokines (Ait-Oufella, Taleb, Mallat, & Tedgui, 2011; Buckley & Ramji, 2015). Transforming growth factor beta 1 (TGF-β1) is a pleiotropic cytokine that can be either pro-atherogenic or antiatherogenic depending on the neighboring conditions (McLaren, Michael, Ashlin, & Ramji, 2011; Ramji & Davies, 2015). The TGF-β1 intervenes in the canonical signaling pathway that mediates the formation of the complex with Smad2 and Smad3 proteins. Any disturbance in the expression of TGF-β1 causes the initiation of important vascular effects that influence the way the atherosclerotic lesion evolves such as fibrosis induction, extracellular matrix (ECM) remodeling and change of vascular smooth-muscle cells (VSMCs) into a pathological phenotype, lipid accumulation followed by stimulating the expression of biglycan, promotion of cell apoptosis, and increase of gene expression of molecules with pro-atherogenic properties (e.g., plasminogen activator inhibitor type 1 (PAI-1) and monocyte chemoattractant protein-1 (MCP-1)). Moreover, it is well acknowledged that target genes activated and regulated by the TGF-β1/Smad2/3 signaling pathway, considered a hallmark of many vascular diseases, encode for type I and III collagen, connective tissue growth factor (CTGF), and ECM, which are key events in the development of vascular intimal hyperplasia (Kalinina et al., 2004; Schiffer, von Gersdorff, Bitzer, Susztak, & Bottinger, 2000).

Numerous animal studies with diet-induced atherosclerosis have been able to provide essential information on understanding the mechanisms underlying this pathology (Georgescu et al., 2016). Many studies have shown that when endothelial cells are activated, they secrete a number of nanovesicles that would help initiate and aggravate the atherogenic process. The term nanovesicles, also known as extracellular vesicles (EVs), bring together two separate classes of small particles of the order of nm called exosomes (40–100 nm) and microparticles (MPs) or microvesicles (MVs) (100–1,500 nm) that are released by cells physiologically, and in pathological conditions this release is amplified (Alexandru et al., 2021; Simionescu, Zonda, Petrovici, & Georgescu, 2021). These vesicles are rich in lipids, proteins, and microRNAs (miRNAs) and have the ability to regulate posttranscriptional gene expression in target cells (Constantin, Filippi, Alexandru, Nemecz, & Georgescu, 2020). The data claim that after their release into the circulation, they would fuse with the target cells using receptor–ligand structures, transferring inflammatory cellular components that originate from the source cells to the recipient cells (Georgescu & Simionescu, 2021). In this way, they become nanocarriers that could also carry a separate class of endogenous miRNAs, affecting gene and protein expression, aggravating the evolution of the pathology as a whole; thus, they can be seen as biomarkers for endothelial dysfunction when their circulating levels are increased (Georgescu et al., 2009; Georgescu et al., 2012).

In this consensus, the ability of vesicles to become messengers of healthy biological information was also observed when released by clinically healthy cells (Alexandru et al., 2017; Alexandru et al., 2020). Also, more and more treatments are being developed on the use of miRNA-based therapeutic strategies in an attempt to modulate and ameliorate their effect. It is known that this complex regulatory network is involved in numerous intracellular signaling pathways disrupting the function of gene and/or several genes that could aggravate and contribute to complications, especially when these miRNAs are overexpressed. Therefore, new approaches to inhibit altered miRNAs could improve and mitigate these changes by restoring normal function. Therapeutic strategies based on targeting short nucleic acids on transcription factors could modulate the transduction of signals in the intracellular network both transcriptionally and posttranscriptionally, inhibiting the expression of target genes involved in inflammation, fibrosis, and vascular remodeling due to atherosclerosis. The most widely used systems today are nonviral lipid and polymer-based siRNA delivery systems called lipoplexes, polyplexes, or liposomes, with dimensions ranging from 100–200 nm, which when administered systemically showed that they have been extensively taken up by the vasculature of the heart. All of these features should be considered for their implementation in clinical trials/application.

According to these data, the use of EVs that are lipid bilayer-delimited particles could serve as exogenous siRNA embedding systems and can be used as RNA interference-based therapy for silencing the genes involved in downstream signaling of pro-inflammatory and pro-angiogenic stimuli (Dorsett & Tuschl, 2004; Kowalski et al., 2011; Youn & Park, 2015). Also, the potential of using a targeted therapy based on Smad2/3 siRNA to block Smad2/3, pivotal downstream effectors in development of atheroma function, could protect against atherosclerosis development.

However, there are not enough data on new approaches by which these noncellular therapeutic agents could detect the first signs of the disease and also the effective way in which they could regress the changes already installed. Currently, the implementation and development of emerging technologies that allow the identification of biomarkers could represent a new stage in the early diagnosis of atherosclerotic CVD. These would be an essential step in how patients can be monitored depending on the prognosis of the disease, the risk of developing complications, and their use for therapeutic purposes.

Based on all these data, we set out to investigate the effects of EVs-based nanotherapeutics on atherosclerosis-induced vascular dysfunction and its key molecular players. Since Smad2/3-mediated TGF-β1/AngII signaling is known to have a decisive role in vascular inflammation and atherogenesis, we decided to block SMAD 2/3 as well. For this purpose, an animal model of atherosclerosis, hypertensive–hyperlipidemic (HH) hamster, was transplanted with EVs (ADSCs) or EVs (MSCs) transfected or not with Smad2/3 siRNA. Generally speaking, EV or the RNA interference technologies are widely applied in the development of experimental therapeutic for many diseases. We analyzed by comparing the effects of EVs (ADSCs) or EVs (MSCs) transfected or not with Smad2/3 siRNA on consequences of atherogenic diet on vascular health.

## Materials and Methods

### Experimental Murine Models With Diet-Induced Atherosclerotic Cardiovascular Disease Transplanted With Extracellular Vesicles (Adipose Tissue Stem Cells) or Extracellular Vesicles (Mesenchymal Stem Cells) Transfected or Not With Smad2/3 siRNA

**FIGURE 1:**
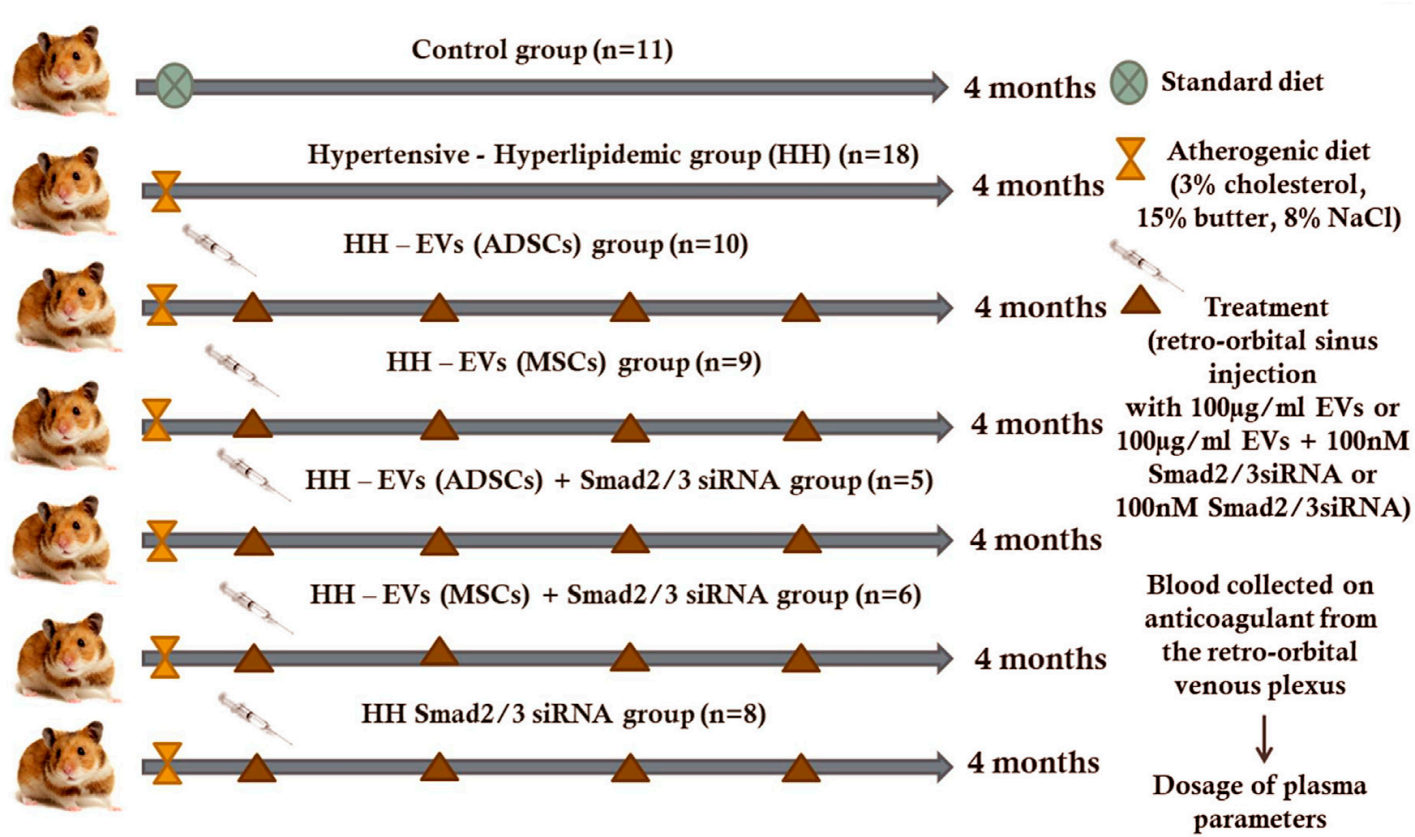
*Schematic representation of experimental animal models obtained for a period of 4 months.* Golden Syrian hamsters (67 males and 3 months old) were divided into seven experimental groups: **(1)** control *(C group)*; **(2)** simultaneously hypertensive–hyperlipidemic *(HH group)*; **(3,4)** HH hamsters with retro-orbital sinus injection containing 100 μg/ml EVs from both ADSCs and BM-MSCs **(*HH-EVs (ADSCs) group and HH-EVs (MSCs) group*))**; **(5,6)** HH hamsters with retro-orbital sinus injection containing 100 μg/ml EVs (from ADSCs or BM-MSCs) transfected with 100 nM Smad2/3 siRNA *(HH-EVs (ADSCs)+Smad2/3 siRNA group and HH-EVs (MSCs)+ Smad2/3 siRNA group)*; **(7)** HH hamsters with subcutaneous injection containing 100 nM Smad2/3 siRNA *(HH-Smad2/3 siRNA group)*. The HH group was obtained by combining the atherogenic and high-salt diet.

A total of 77 male golden Syrian hamsters, 3 months of age, 117.8 ± 2.81 g body weight, were divided into seven experimental groups: normal healthy animals fed with standard food containing basal 1%NaCl for 4 months (*C group*); simultaneously, hypertensive–hyperlipidemic animals generated by combining two feeding conditions (the standard chow enriched with 3% cholesterol and 15% butter for hyperlipemia and 8% NaCl gavage for hypertension) administered daily for 4 months (*HH group*); HH animals transplanted with 100 μg/ml EVs (ADSCs) or EVs (MSCs) obtained from healthy hamsters, transfected or not with 100 nM Smad2/3 siRNA, by using retro-orbital sinus injections (in a volume of 300 µL phosphate-buffered saline (PBS)) once a month for 4 months, starting with the second week of the atherogenic diet (*HH-EVs (ADSCs), HH-EVs (MSCs)*, *HH-EVs (ADSCs)+ Smad2/3 siRNA*, and *HH-EVs (MSCs)+ Smad2/3 siRNA* groups); HH animals treated with Smad2/3 siRNA (100 nM in a volume of 300 µL PBS) via subcutaneous injections administrated once a month for 4 months, starting with the second week of the atherogenic diet (*HH-Smad2/3 siRNA group*) (Figure 1).

All experimental groups of hamsters thus obtained were kept in the same housing conditions at 25°C with a 12/12 h light/dark cycle. At the end of the 4 months (16 weeks) of the diet, hamsters were weighed to assess body weight and anesthetized with 2% isoflurane for blood collection. Subsequently, they were sacrificed under anesthesia (a mixture of 80 mg ketamine, 10 mg xylazine, and 2 mg acepromazine/kg body weight in a sterile isotonic saline (0.9% saline)), perfused with PBS containing 1 mM CaCl_2_ for tissues blood removal, in order to collect the organs of interest (thoracic aorta and carotid arteries for biochemical, structural, and functional assays). All the protocols for animal use were approved by the Ethics Committee from the Institute of Cellular Biology and Pathology “Nicolae Simionescu” according to Decision No.11/08.08.2017 and National Sanitary Veterinary and Food Safety Authority (Bucharest, Romania) in compliance with Project Authorization No. 575/13.11.2020. Also, all the experiments on animals were conducted in accordance with the Guide of the Care and Use of Laboratory Animals published by the US National Institutes of Health (NIH publication no. 85–23, revised 1996) and were conducted in accordance with national, European, and international legislation on the use of experimental animals in biomedical research.

#### Isolation and Characterization of Adipose Tissue Stem Cells and BM-Mesenchymal Stem Cells

In order to obtain ADSCs and BM-MSCs, two main tissue sources were used: subcutaneous adipose tissue and hematogenous bone marrow tissue from the tibia and femoral marrow compartments, harvested from 50 hamsters of healthy origin. The cells obtained in the primary culture were kept in an incubator with 5% CO_2_ and maintained at 37°C until they reached confluence in DMEM/F-12 (Dulbecco’s modified Eagle medium/nutrient mixture F-12) (Gibco) containing 10% fetal bovine serum (FBS) (Gibco) for ADSC and DMEM, high glucose, GlutaMAX™ Supplement, and pyruvate (Gibco) with 10% FBS for BM-MSC, and both media being supplemented with 1% antibiotics (mix of penicillin, neomycin, and streptomycin). The percentage of cell viability was evaluated by trypan blue exclusion assay. When culture reached about 90% confluence (within 2–3 days), cells were treated with 0.25% trypsin and subcultured at a split ratio of 1:3. The cells at passage three were used for the specific characterization. The presence of stem cell markers, namely CD73, CD90, CD29, CD44, CD117 (antibodies from R&D Systems), and CD105 (antibody from BioLegend) and the absence of markers of hematopoietic cells, namely CD45 (antibody from BioLegend), CD14, and CD31 (antibodies from R&D Systems) were analyzed by flow cytometry.

#### Obtaining and Characterization of Extracellular Vesicles (Adipose Tissue Stem Cells) and Extracellular Vesicles (Mesenchymal Stem Cells)

The MSCs (ADSCs or BM-MSCs) at passage five were kept in a serum-free medium for 48 h in order to release EVs (ADSCs) or EVs (MSCs). Repeated centrifugations and ultracentrifugations were performed on the cell-conditioned medium for EV isolation. Centrifugation at 2,500 *g* for 10 min was used to remove cellular debris and 16.000 x g for 5 min to remove apoptotic bodies. After that, the supernatant was subjected to ultracentrifugation at 100.000 x g for 20 h at 4°C to obtain EVs (microvesicles (MVs or MPs) and exosomes) in the pellet. Then, the EVs obtained in the pellet were washed with sterile PBS at 100.000 x g for 2 h and 30 min at 4°C. Finally, the pellet containing EVs was resuspended in 500 μL sterile PBS and maintained at −80°C until further analysis. To highlight the presence of both exosomes (50–100 nm) and microvesicles (100–1,000 nm), the particle size was assessed using a Malvern Zetasizer Nano ZS. The characterization of EVs isolated from the culture medium from both ADSCs and BM-MSCs was performed by flow cytometry following the presence of the markers CD63^+^ (for exosomes) (Thermo Fisher) and Annexin V^+^ (for microvesicles) (SantaCruz Biotechnology, Inc.). To evaluate the accuracy and repeatability of EV size, a comparative method, namely transmission electron microscopy (TEM, FEI Talos F200C) was employed according to the protocol described by Sorop et al., (2020). In brief, negative staining for TEM was performed on carbon-coated copper grids (100 mesh; Agar Scientific). A volume of 5 μL of sample containing EVs was incubated for 2 min on grids at room temperature (RT) and stained with 2% uranyl acetate. Image acquisition was performed at RT using a 200 kV Talos F200C TEM (Thermo Fisher Scientific).

#### Transfection of Extracellular Vesicles With Smad2/3 siRNA

Lipofectamine™ RNAiMAX transfection reagent (Thermo Fisher Scientific) was used to transfect EVs with a siRNA-targeting Smad2/3 (100 nM final concentration) (Santa Cruz Biotechnology, Inc.) according to the manufacturer’s protocol. In brief, two different mixtures were made, one consisting of 3 μL Smad2/3 siRNA and 50 μL OPTI-MEM (Gibco by Life Technologies) and the other of 3 μL Lipofectamine and 50 μL OPTI-MEM, which were later homogenized. A volume of 100 μg/ml EVs (ADSCs) or EVs (MSCs) was added to the resulting mix, incubated for 72 h at 37°C in 5% CO_2_ atmosphere, and ultracentrifuged at 100.000xg for 2 h at 4°C. The resulting pellet containing 100 μg/ml transfected EVs was resuspended in 300 μL PBS. The efficiency of EV transfection was verified with siRNA-FITC, used as a positive control, by flow cytometry.

### Analysis of the Main Plasma Parameters of Experimental Models

The plasma samples were obtained from all experimental animal groups by collecting about 1 ml venous blood from the retro-orbital plexus in vacutainers containing EDTA (EDTA-treated tubes) and centrifuging at 2,500 x g for 10 min at 4°C. This procedure was performed monthly for 4 months of follow-up under light anesthesia with 2% isoflurane (inhalation vapor: Isoflutek 1000 mg/g) with pre-weighing of animals under fasting conditions.

The plasma levels of total cholesterol, HDL-cholesterol, LDL-cholesterol, triglycerides, and glucose were determined by a colorimetric method using commercially available kits from DIALAB GmbH, Vienna, Austria. The samples were made in duplicate and measured at a wavelength between 500–600 nm (Tecan Infinite M200 PRO).

### Echocardiographic Evaluation of the Arterial Wall Structure

For this analysis, the high-resolution ultrasonic imaging system for small animals (Vevo2100) was used. Prior to the procedure, the hamster fur on the chest was removed using an electric hair clipper so as not to interfere with the signal from the device. The animals were anesthetized throughout this diagnostic procedure with 2% isoflurane and placed on a heated platform to keep their body temperature constant. Vital signs (heart rate and pulse) were constantly monitored. Transthoracic echocardiography consisted of parasternal sections on the long axis to determine the thickness of the vascular wall and the inner diameter recorded in the B mode (two-dimensional). The B mode is a major method that helps to assess the anatomical and functional characteristics of the heart and blood vessels. With the help of the M mode, flat sections were obtained, and the diameter in systole and diastole could be measured. For large blood vessels such as the thoracic aorta, VTI (velocity time integral) and VEL (blood velocity) were measured, and the records being obtained in the pulsed wave (PW) Doppler mode that analyzes the hemodynamic characteristics of blood flow. The images were stored in the ultrasound system hard-drive and transferred to an external memory hard-drive for offline analysis. The processing of the images obtained from all experimental animal groups was performed with the help of VevoLab300 software. Echocardiographic evaluation was performed at the end of 4 months of experimental diet/treatment, and the results were compared with the C group.

### Myographic Analysis of Functional Responses of Isolated Blood Vessels

Vascular dysfunction was analyzed by a myograph technique (device that investigates vascular reactivity) used to explore the ability of blood vessels (thoracic aorta and carotid artery) to contract or relax. The blood vessels that were taken for myographic analysis were not previously washed with PBS with CaCl_2_ to avoid endothelial damage. The isolated arterial segments were mounted on the Multi Myograph System-model 620 M in combination with the Automatic Buffer Filler System-625FS (Danish Myo Technology, DMT). For these experiments, ACh (acetylcholine) was used to induce vessel relaxation and NA (noradrenaline), to induce vessel contraction. In order to record the physiological activity of smooth muscle cells (SMCs), the blood vessels (∼200 µm diameter) were cleaned off lipids, cut under a microscope to a length of 3 mm, mounted on the wire-myograph chamber, and immersed in HEPES sodium salt buffer, pH = 7.45 at 37°C. The optimal operating diameter was determined and calibrated, and the standard start procedure was applied to verify the integrity of the mounted blood vessels and the presence of the intact endothelium, by stimulation with 3 × 10^−7^ M NA. Throughout the experiment, O_2_ was constantly bubbled into the myograph’s organ chamber to maintain the function of the blood vessels. Subsequently, the responses of blood vessels in the presence of curves of increasing concentrations of NA or ACh were recorded in real time. The concentration curve used was as follows: 10^–8^ M, 3 × 10^−8^ M, 10^–7^ M, 3 × 10^−7^ M, 10^–6^ M, 3 × 10^−6^ M, 10^–5^ M, 3 × 10^−5^ M, and 10^–4^ M for both NA and ACh. The forces developed by the blood vessels in the presence of the vasoconstrictor or vasodilator agonists were recorded at an interval of 2 min. Finally, the wire tension (mN/mm) developed by the blood vessels in the presence of the vasoconstrictor NA and the relaxation in the presence of the vasodilator ACh, calculated as a percentage (%) of the maximum precontraction at NA, were evaluated. Data acquisition was performed using a PowerLab 4/26 hardware (ADInstruments) and images recorded with LabChart 7 (multichannel chart recorder) software.

### Analysis of the Plasma Ang II and TGF-β1 Levels

After 4 months of the hyperlipemic–hypertensive diet, the blood collected on anticoagulant from the retro-orbital venous plexus was centrifuged at 2,500 x g for 10 min at 4°C to obtain plasma. Subsequently, plasma angiotensin II (Ang II) and transforming growth factor beta 1 (TGF-β1) levels were determined using the commercial Angiotensin II EIA Kit (Sigma-Aldrich) and Human/Mouse TGF-beta 1 Uncoated ELISA Kit (Invitrogen) according to the manufacturer’s protocol. The samples were read on the spectrophotometer (Tecan Infinite M200 PRO) at a wavelength suggested by the manufacturer (450 nm).

### Immunofluorescence Staining and Image Analysis for Detection of Inflammatory Markers

After perfusion by ventricular puncture, the interest organs including thoracic aorta and carotid arteries were excised, immersed in 2% paraformaldehyde (PFA) cryoprotection solution in 0.1 M phosphate buffer, and left at 4°C overnight. Subsequently, the samples were washed with phosphate buffer, subjected to consecutive baths of glycerol of different concentrations (5% for 15 min, 10% for 1 h, 20% overnight, and 50% for 1 h, at 4°C), and kept in the freezer (−20°C) until their processing. To perform the histological sections, the samples were washed (successive washes with 3% sucrose solution in phosphate buffer, 6x for 15 min) and immersed for 30 min in OCT (Tissue-Tek, Sakura). After 30 min, the samples were removed from the OCT, immersed for 30–45 s in liquid nitrogen, and placed in the cryotome (Leica CM1850), where they were mounted on the cutting support. The tissue block was mounted on the mold, and cryosections (5 µm thickness) cut using a microtome blade (MX35 Ultra, Thermo Scientific) were attached to glass slides (Superfrost Plus from Thermo Scientific treated with poly-l-lysine for a better adhesion of the sections on the support), incubated for 30 min at 37°C, and stored at 4°C until use. For immunostaining, the following essential steps were completed: defrosting sections at room temperature (RT), fixing with cold methanol (−20°C), incubation with NaBH_4_ for 1 h at 4°C to reduce tissue autofluorescence, permeabilization with 0.2% Triton X-100 in PBS containing 0.05% Tween 20 (AppliChem) for 30 min at RT, encircling sections on the slide with a lipid pen (Invitrogen), and blocking nonspecific sites with 10% goat serum (Invitrogen). The sections were incubated overnight at 4°C with the following primary antibodies (diluted in PBS with 1% BSA (bovine serum albumin)) used for: COL1A (1:250, Santa Cruz Biotechnology), α-SMA (1:200, Cell Signaling Technology), Cx43 (1:200, Thermo Fisher Scientific), MMP-2 (1:200, Santa Cruz Biotechnology), VCAM-1 (1:200, Santa Cruz Biotechnology), CD3e^+^ (1:200, Thermo Fisher Scientific), CD68^+^ (1:200, Santa Cruz Biotechnology), and MHC-II^+^ (1:250, Thermo Fisher Scientific). Then, the sections were washed three times with PBS, incubated with the secondary antibody labeled with Alexa Fluor 568 goat anti-rabbit IgG (H + L) 1:1,000 or Alexa Fluor 647 donkey anti-mouse IgG (H + L) 1: 500 or Alexa Fluor 488 goat anti-rabbit IgG (H + L) 1:500, and washed three times with PBS. Subsequently, DAPI solution (5 mg/ml in 10 mM PBS) was added for 5 min to label the nuclei, and sections were washed three times with PBS. Finally, a liquid mountant (ProLong, Invitrogen) was applied directly to fluorescently labeled tissue samples on microscope slides.

In separate experiments, the ROS (reactive oxygen species) expression level was analyzed by adding 6 µM DHE (fluorochrome dihydroethidium, Sigma-Aldrich) on tissue sections for 30 min at RT. The images were captured and analyzed under an inverted fluorescent microscope (Axio Vert. A1 Fl, Carl Zeiss, software Axio Vision Rel 483SE64-SP1), under ×20 magnification. Image analysis was performed with the help of the ImageJ program.

### Protein Expression Evaluation by Western Blot Analysis

#### Protein Extraction

For the protein extraction, RIPA buffer (Thermo Scientific) containing 100 mM PMSF, a cocktail of phosphatase B inhibitors and protease inhibitors, was added to the tissue (thoracic aorta and carotid artery). The samples were minced into very small pieces using scissors and homogenized at 5,000 rpm x five rounds for 2 min each, using the Minilys Personal High Power Tissue Homogenizer (Bertin Technologies) and glass beads with 1 mm diameter. After processing, the tubes were left in the refrigerator overnight, centrifuged the next day at 15600 rpm, for 5 min, at 4°C, and then the supernatant containing the protein lysate was transferred to new tubes and stored at -20°C until the protein concentration was dosed. To determine the protein concentration, BCA Protein Assay Kit (ThermoScientific) was used. The samples were read in duplicate at an absorbance of 562 nm at a detection range of 20–2000 μg/ml and reported on a standard curve of BSA.

#### Gel Electrophoresis and Immnunoblotting

The samples (100 μg/lane for the thoracic aorta and 50 μg/lane for the carotid artery) were separated by SDS-PAGE gels and subsequently electrophoretically transferred to a nitrocellulose membrane. A wide range molecular weight marker (6.5÷200 kDa) (Sigma) was loaded into one lane as a standard. Prior to antibody labeling, the nitrocellulose membrane was immersed in a nonspecific site blocking solution (TBS solution with 3% BSA (Applichem) or TBS solution with 5% Blotto, nonfat dry milk (Santa Cruz Biotechnology)), then incubated with primary monoclonal antibodies: β-actin–used as a loading control (1:200, SantaCruz Biotechnology), ATF2 (1:500, Thermo Fisher Scientific), SMAD2/3 (1:1,000, Thermo Fisher Scientific), pSMAD2/3 (1:1,000, Thermo Fisher Scientific), and NF-kB p50/NF-kB p65 (1:200, SantaCruz Biotechnology), which recognize the protein of interest. Incubation with primary antibodies was performed overnight at 4°C. Washing extensively was followed to remove unbound antibodies, and incubation with secondary antibodies coupled with horseradish peroxidase (HRP), anti-mouse antibodies (1:2000, Thermo Fisher Scientific), or anti-rabbit antibodies (1:5,000, Thermo Fisher Scientific), which specifically recognize primary antibodies. Incubation with secondary antibodies was performed for 1 h at RT. Detection of intensity of bands was made in the presence of ECL (enhanced chemiluminescent substrate for HRP detection), and the TotalLab program was used to quantify the information. The investigated protein bands were reported to the band corresponding to β-actin, a housekeeping reference protein.

### Ribonucleic Acid Extraction and Quantification of miRNAs by Real-Time Quantitative Polymerase Chain Reaction Analysis

Total RNA was extracted from cryopreserved tissue (thoracic aorta and carotid artery) using miReasy Micro Kit (QIAGEN). In brief, the tissue was weighed (∼10 mg) and crushed very well using fine scissors. Over the tissue fragments, 1.4 mm zirconium oxide beads (Precellys) and 700 μL lysis buffer (QIAzol Lysis Reagent) were added. The samples were grounded at 1800rpm, 6x for 1 min on ice, placed for 10 min at −20°C, left for 10 min at RT, centrifuged at 10,000 x g for 5 min at 4°C, and the supernatant was processed according to the manufacturer’s protocol. Finally, RNA was eluted in 16 μL RNase-free water. The RNA purity and concentration were evaluated by spectrophotometry using NanoDrop 2000c (Thermo Fisher) and kept at −80°C until examinations. The reverse-transcription of RNA was performed using TaqMan MicroRNA Reverse Trascription Kit (Applied Biosystems) in combination with TaqMan-Gene Expression Master Mix according to the instructions of the manufacturer on a Veriti real-time PCR system (Applied Biosystems). The study of miRNA expression was carried out on six miRNAs: hsa-miR-21(ID:0,000,397), hsa-miR-192 (ID:000,491), hsa-miR-200b (ID:002,251), hsa-miR-29a (ID:002,112), hsa-miR-210 (ID:000,512), and hsa-miR-146a (ID:000,468). Each reaction was performed in triplicate. The miRNA expression level was normalized to U6 small nucleolar RNA snRU6 (ID:00,197) and quantified using the 2^**–∆∆Ct**^ calculation method. The VIIA7 software v1.2 (Applied Biosystems) with the automatic quantification cycle (Cq) setting was used to analyze the data according to the protocol described by Alexandru et al., (2017); Alexandru et al., (2019); Alexandru et al., (2020).

## Results

### Characterization of Adipose Tissue Stem Cells and BM-Mesenchymal Stem Cells Revealed the Presence of Stem Cell-Specific Markers

The analysis of cells at passage three by flow cytometry was able to validate the phenotype of the two MSC types, some isolated from subcutaneous adipose tissue called ADSCs and others from bone marrow aspirate called BM-MSCs. As expected, the results showed that both cell types displayed specific markers for stem cells, namely CD73, CD90, CD105, CD29, CD44, and CD117 but were negative for specific markers of hematopoietic cells, namely CD45, CD14, and CD31 (the results are not shown, but some of them are found in the article published by Constantin et al., 2017).

### Characterization of Extracellular Vesicles From the Secretome of Adipose Tissue Stem Cells or BM-Mesenchymal Stem Cells Disclosed the Existence of Both Microvesicles and Exosomes

**FIGURE 2:**
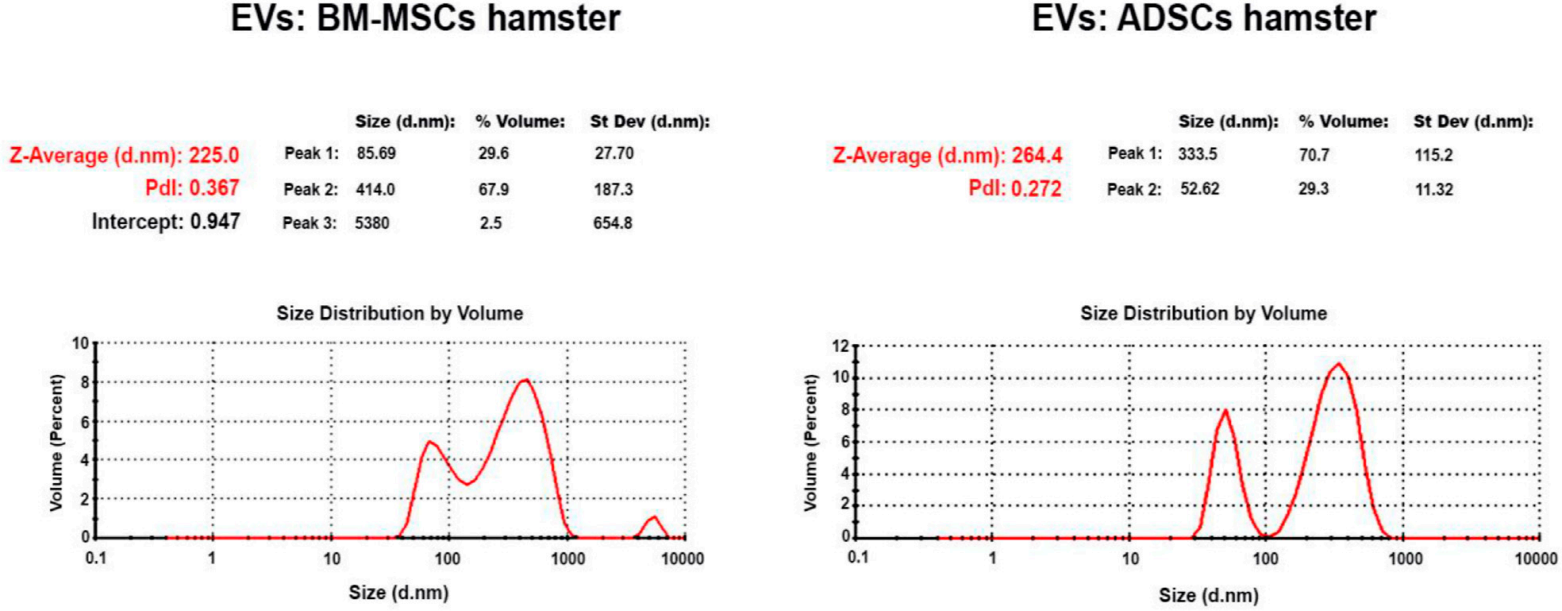
Particle size distribution of EVs secreted in the culture medium by ADSCs and BM-MSCs showed two different populations represented by the two peaks; the first peak at the small size range (∼50–100 nm) specific to exosomes and the second at the high-size range (∼100–1000 nm) specific to microvesicles (*Y*-axis represents the number of EVs, and the *X*-axis represents the size of EVs).

EVs were isolated from the secretome of healthy hamster adipose tissue-derived stem cells (ADSCs) or of bone marrow-derived stem cells (BM-MSCs) and kept for 48 h in media without serum. The EVs thus obtained (EVs (ADSCs) and EVs (MSCs)) were analyzed by Zetasizer Nano ZS ZEN3600 to establish their size (Figure 2). The distribution curves of the EV dimensions are bimodal, both in the case of EVs (ADSCs) and EVs (MSCs), and the values of particle dimensions (Z) being between 50 and 100 nm for exosomes (∼30%) and between 100 and 1,000 nm for microvesicles (∼70%) (Figure 2).

**FIGURE 3:**
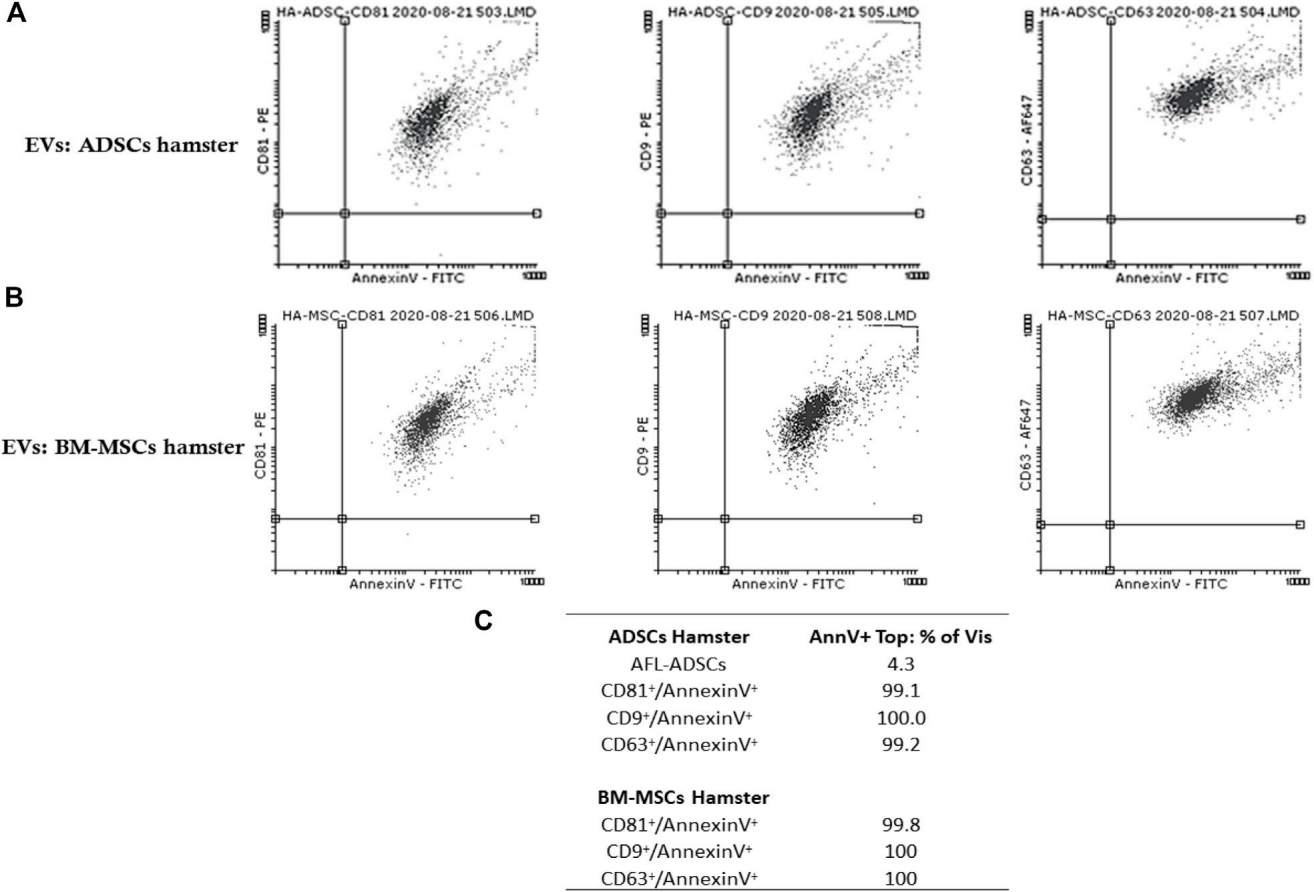
Detection and characterization by flow cytometry of EVs isolated from the culture medium from ADSCs/BM-MSCs of healthy hamsters highlight the presence of both exosomes (CD63^+^, CD9^+^, and CD81^+^) and microvesicles (AnnexinV^+^). **(A)** Representative dot plots double-stained with purified EVs from ADSC fraction with CD81^+^ (labeled with PE)/Annexin V^+^ (labeled with FITC), CD9^+^ (labeled with PE)/Annexin V^+^ (labeled with FITC), and CD63^+^ (labeled with AF647)/Annexin V^+^ (labeled with FITC); **(B)** representative dot plots double-stained with purified EVs from the BM-MSC fraction with CD81^+^ (labeled with PE)/Annexin V^+^ (labeled with FITC), CD9^+^ (labeled with PE)/Annexin V^+^ (labeled with FITC), and CD63^+^ (labeled with AF647)/Annexin V^+^ (labeled with FITC) **(C)** Percentage of EV specific markers for both exosomes and microvesicles.

Flow cytometry analysis confirmed the presence in the purified EV fraction (EVs(ADSCs) and EVs (MSCs)) of both exosomes (CD63^+^, CD9^+^, and CD81^+^) and microvesicles (AnnexinV+) (Figures 3A–C).

**FIGURE 4:**
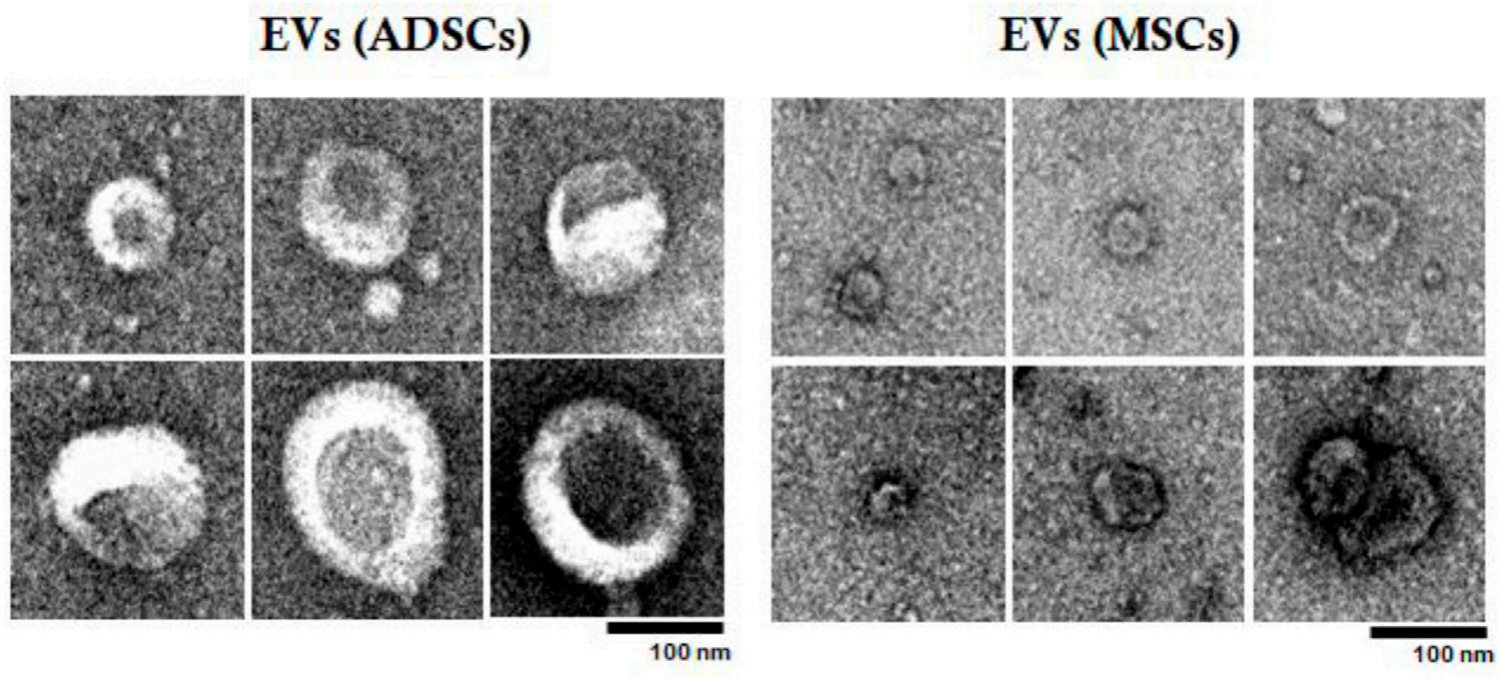
Examination of EVs (ADSCs) and EVs (MSCs) by transmission electron microscopy (TEM). EVs were collected from the secretome of healthy hamster adipose tissue-derived stem cells (ADSCs) or of bone marrow-derived stem cells (BM-MSCs). The effect of atherogenic diet and treatment on main plasma parameters were investigated.

Subsequently, EVs were examined by TEM to confirm their size and characterize them from a structural point of view (Figure 4). Analysis by TEM showed that these EV fractions obtained from both ADSCs and BM-MSCs contain heterogenous particles of larger or smaller dimensions in a range of values specific to exosomes and microvesicles. These particles have a typical cup-shaped structure with a membrane and cytoplasm. Analyzing by comparison between EVs (ADSCs) and EVs(MSCs), it was observed that EVs(ADSCs) are larger than EVs(MSCs), meaning that in the fraction of EVs the percentage of microvesicles is higher than that of the exosomes (Figure 4).

The seven investigated experimental animal groups (*C*, *HH*, *HH-EVs(ADSCs)*, *HH-EVs(MSCs)*, *HH-EVs(ADSCs)+Smad2/3siRNA*, *HH-EVs(MSCs)+Smad2/3siRNA*, and *HH-Smad2/3siRNA*) were evaluated for body weight and plasma parameters (glucose, total cholesterol, HDL-cholesterol, LDL-cholesterol, and triglycerides) at the beginning of the experimental period and the end of each month over the 4-month experimental period, while they were on an atherogenic diet (food enriched with 15% butter and 3% cholesterol) and received daily gavage with 8% NaCl.

The weighing of the hamsters revealed a slight insignificant decrease at two, three, and four months of diet in the case of hamster in the HH group, while in the control hamsters, the weight was constant throughout the experimental period. The treated groups showed a slight weight gain, especially in the HH-Smad2/siRNA and HH-EVs (MSCs) groups (Table 1).

**TABLE 1:** Clinical characteristics and biochemical parameters of the seven experimental animal groups, control (C); simultaneously hypertensive–hyperlipidemic (HH); HH-EVs(ADSCs), HH-EVs(MSCs), HH-EVs(ADSCs)+Smad2/3siRNA, HH-EVs(MSCs)+Smad2/3siRNA, and HH-Smad2/3 siRNA, after 16 weeks of standard diet or atherogenic diet, and at 14 weeks post-treatment with EVs (ADSCs) or EVs (MSCs) transfected or not with Smad2/3siRNA during the diet-induced atherosclerotic process. Data are means ± SD of duplicate determinations. The statistical significance, noticeably different, was represented as ***p < 0.005, **p < 0.01, *p < 0.05 values versus control group and ^###^ *p* < 0.005, ^##^ *p* < 0.01, ^#^ *p* < 0.05 values versus HH group. Two-way ANOVA and Bonferroni post-test were applied.

**After 16 weeks of standard diet/atherogenic diet**	**Control (*n* = 11)**	**HH (*n* = 18)**	**HH-EVs (ADSCs) (*n* = 10)**	**HH-EVs (MSCs) (*n* = 9)**	**HH-EVs (ADSCs) + Smad2/3 siRNA (*n* = 5)**	**HH-EVs (MSCs) + Smad2/3 siRNA (*n* = 6)**	**HH-Smad2/3 siRNA (*n* = 8)**
**Initial body weight (g)**	117.8 ± 2.81	125.9 ± 3.90	122.5 ± 3.93	132.78 ± 5.51	132.17 ± 7.3	147.33 ± 6.82	128.11 ± 5.62
**Final body weight (g)**	116 ± 3.39	99.38 ± 2.76	110.9 ± 5.97	121.55 ± 6.07	105.33 ± 4.74	110.33 ± 8.36	120.5 ± 3.65
**Glycemia (mg/dl)**	116.02 ± 22.69	137.76 ± 48.25	137.97 ± 19.16	152.5 ± 21.81	108.81 ± 29.49	125.2 ± 16.1	133.96 ± 9.42
**Cholesterol (mg/dl)**	128.04 ± 14.47	641.66 ± 26.13 (***p < 0.005)	244.45 ± 60.6 (^###^ *p* < 0.005)	300.83 ± 60.77 (*p < 0.05) (^###^ *p* < 0.005)	208.22 ± 37.7 (^###^ *p* < 0.005)	267.1 ± 64.12 (^###^ *p* < 0.005)	201.7 ± 36.47^###^ *p* < 0.005)
**Triglycerides (mg/dl)**	93.42 ± 4.17	1,073 ± 6.87 (***p < 0.005)	212.26 ± 3.42 (^###^ *p* < 0.005)	175.43 ± 5.62 (^###^ *p* < 0.005)	202.82 ± 4.89 (^###^ *p* < 0.005)	217.31 ± 6.87 (^###^ *p* < 0.005)	652.22 ± 55.75 (***p < 0.005) (^###^ *p* < 0.005)
**HDL cholesterol (mg/dl)**	54.44 ± 6.47	109.73 ± 49.59 (**p < 0.01)	89.71 ± 29.72 (*p < 0.05)	82.67 ± 25.27	97.41 ± 3.97	90.24 ± 25.77	91.43 ± 23.52
**LDL cholesterol (mg/dl)**	73.67 ± 4.69	635.43 ± 8.8 (***p < 0.005)	307.09 ± 7.23 (***p < 0.005) (^##^ *p* < 0.01)	303.18 ± 8.4 (***p < 0.005) (^##^ *p* < 0.01)	280.26 ± 2.3 (**p < 0.01) (^##^ *p* < 0.01)	364.18 ± 5.24 (***p < 0.005) (^##^ *p* < 0.01)	284.94 ± 43.91 (***p < 0.005) (^##^ *p* < 0.01)

Regarding the biochemical parameters, compared to the control group, the hamsters from the HH group showed a significant increase in plasma concentrations of total cholesterol (5.01 times), LDL-cholesterol (8.62 times), and triglycerides (11.48 times) throughout the experimental period. For HDL-cholesterol, the values for HH group remained constant in the first 2 months of the diet, while in the third and fourth month they began to grow, registering a statistically significant increase in the fourth month of the diet (2.01 times) (Table 1). In addition, blood glucose values did not differ significantly between the seven experimental groups, and the values being relatively constant during the 4 months of diet and treatment (Table 1).

Concerning the effects of the treatments, both EVs and EVs transfected with Smad2/3siRNA treatment led to a significant reduction in the levels of total cholesterol (2.62 times for EVs (ADSCs) and 2.13 times for EVs (MSCs)), triglycerides (5.05 times for EVs (ADSCs) and 6.11 times for EVs (MSCs)), and LDL-cholesterol (2.06 times for EVs (ADSCs) and 2.09 times for EVs (MSCs)). The combined administration of EVs and Smad2/3 siRNA had a beneficial effect on plasma parameters significantly decreasing their levels compared with those in the HH group. The values were comparable to those obtained in the case of single therapy with EVs (ADSCs) or EVs (MSCs) (Table 1). Treatments based on EVs (ADSCs) and EVs (MSCs) alone or in combination with Smad2/3siRNA and administration of Smad2/3siRNA alone had a beneficial effect on plasma parameters reducing the levels compared with those in the HH group. HH-Smad2/3siRNA is the only experimental group that recorded a significant increase in triglyceride levels compared to both the control and the other treated groups (Table 1).

It was also observed that following the administration of the atherogenic diet, the liver was affected, having a yellow and greasy appearance, which indicates the presence of hepatic steatosis, and the plasma had a milky, dense consistency due to the accumulation of a large amount of lipids in the bloodstream. A visible improvement of the appearance of the liver and blood was noticed in the groups of treated animals. It is important to mention that the significant change in the lipid profile, a risk factor correlated with the development of atherosclerosis, proves that hamsters in the HH group are dyslipidemic. Our previous results have shown that this group of hamsters (HH) also presented significant increases in systolic and diastolic blood pressure and heart rate (Georgescu et al., 2012), which suggest that the HH group is also hypertensive. These cumulative results prove that the HH group is hypertensive–hyperlipidemic, which means that the experimental model, the hypertensive–hyperlipemic hamster, was successfully obtained.

### Echocardiographic Evaluation Revealed the Vascular Structural and Blood Flow Changes Induced by the Atherogenic Diet; the Stem Cell-Derived Extracellular Vesicle-Based Treatment Produced a Noticeable Improvement in Them

**FIGURE 5:**
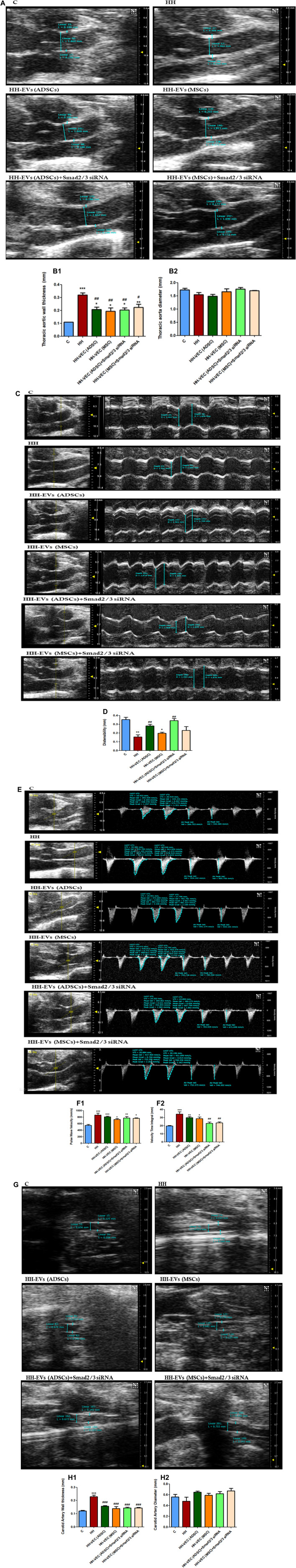
Changes in the blood flow and structure of the thoracic aorta and carotid artery were isolated from the investigated experimental groups (C, HH, HH-EVs(ADSCs), HH-EVs(MSCs), HH-EVs(ADSCs)+Smad2/3siRNA, and HH-EVs(MSCs)+Smad2/3siRNA) as a measure of vascular rigidity. **(A)** Representative B-mode recordings, which highlight the wall thickness and the inner diameter of the thoracic aorta; **(B)** graphical representation of wall thickness (mm) **(B.1)** and inner diameter (mm) **(B.2)** in the case of the thoracic aorta; **(C)** representative records obtained in M-mode, which highlight the diameter in systole and diastole of the thoracic aorta; **(D)** graphical representation of thoracic aortic distensibility (mm); **(E)** representative recordings obtained in pulsed Doppler-mode, which highlight the velocity time integral (VTI) and velocity (Vel) of the thoracic aorta; **(F)** graphical representation of velocity (mm/s) **(F.1)** and velocity time integral (mm) **(F.2)** at the level of the thoracic aorta; **(G)** representative B-mode recordings, which highlight the wall thickness and the inner diameter of the carotid artery; **(H)** graphical representation of wall thickness (mm) **(H.1)** and inner diameter (mm) **(H.2)** in the case of the carotid artery. Data are shown as the mean ± SD of each experimental group after 4 months of diet and treatment. The statistical significance, noticeably different, is represented as ***p < 0.005, **p < 0.01, *p < 0.05 versus control group and ^###^ *p* < 0.005, ^##^ *p* < 0.01, ^#^ *p* < 0.05 versus HH group. The values were calculated by two-way ANOVA and Bonferroni post-test.

To determine whether the atherogenic diet affects vascular integrity, we investigated the structure and function of the thoracic aorta and carotid artery by echocardiography. First, we followed the thickness of the vascular wall and the inner diameter of the thoracic aorta using B-mode recordings. Thickening of the vascular wall was observed in group HH compared to control group (Figures 5A, B1). In all groups that received treatment (HH-EVs(ADSCs), HH-EVs(MSCs), HH-EVs(ADSCs)+ Smad2/3siRNA, and HH-EVs(MSCs)+ Smad2/3siRNA), a significant decrease in the thickness of the vascular wall was observed compared to that of the HH group, meaning that this treatment improves the functionality of the blood vessels (Figures 5A, B1). Regarding the inner diameter, no significant changes were observed between all investigated experimental groups (Figures 5A, B2). Through the recordings in M-mode, we followed the diameter in systole and diastole. The difference between the diameter in systole and that in diastole provides information on the elasticity of the blood vessel. The HH group recorded significantly lower values than the control group, while in the two treated groups an improvement was observed in terms of blood vessel distensibility (Figures 5C,D). The groups that received treatment based on EVs (ADSCs) or EVs (ADSCs) transfected with Smad2/3siRNA had a better result in restoring the elasticity/distensibility of the arterial wall (Figures 5C,D). Quite the opposite, the treatment based on EVs from MSCs did not bring any noticeable enhancements (Figures 5C,D). Through the recordings in the pulsed Doppler-mode, we followed two characteristic parameters for the hemodynamics of the blood flow–velocity time integral (VTI), which represents the length of the ejection tract measured in mm, and velocity (Vel) (blood flow rate measured in mm/s) at the thoracic aorta. The analysis of the images showed that the HH group registered significantly increased values (***p < 0.005) for both Vel and VTI correlated with the structural changes in the arterial wall and the increase in blood pressure triggered after daily administration of gavage with 8% NaCl, for 16 experimental weeks compared to the control group (Figures 5E, F1,F2). The groups of treated animals (HH-EVs(ADSCs), HH-EVs(MSCs), HH-EVs(ADSCs)+Smad2/3siRNA, and HH-EVs(MSCs)+Smad2/3siRNA) showed significantly lower values for VTI than HH, especially those transfected with Smad2/3siRNA (Figures 5E, F1). The measurements that characterize velocity showed that there is no difference between the treated groups, only a slight decrease compared to the HH group, without statistical significance (Figures 5E, F2). The VTI and Vel are two important parameters that give not only an image of the global systolic function but also of the structural and functional changes at the level of the isolated thoracic aorta from all investigated groups. The increase of these parameters suggests the presence of structural alterations and the debut of vascular dysfunction in animals in the HH group. Another investigated blood vessel was the carotid artery. Echocardiographic recordings in the B-mode showed significantly increased values of vascular wall thickness in the HH group (***p < 0.005) compared to that in the healthy animals, accompanied by a slight decrease in inner diameter (Figures 5G, H1,H2). As for the animals that received EV-based treatment (HH-EVs(ADSCs), HH-EVs(MSCs), HH-EVs(ADSCs)+Smad2/3siRNA, and HH-EVs(MSCs)+Smad2/3siRNA), they had significantly reduced values in the thickness of the vascular wall compared to that of the HH group (###*p* < 0.005), showing that the treatment improves the vascular structure in smaller vessels as well (Figures 5G, H1). No visible and statistical difference in the thickness of the vascular wall was observed between the groups of treated animals (Figures 5G, H1). In terms of inner diameter, no statistically significant differences were found between the investigated experimental animal groups: C; HH; HH-EVs(ADSCs), HH-EVs(MSCs), HH-EVs(ADSCs)+Smad2/3siRNA, and HH-EVs(MSCs)+Smad2/3siRNA (Figures 5G, H2). This was because the carotid artery is a small blood vessel, and we could not follow the other structural and functional changes by echocardiography.

### Vascular Myography Showed Altered Functional Responses of Blood Vessels After 4 months of Atherogenic Diet; Favorable Effects of Treatment With Extracellular Vesicles (Adipose Tissue Stem Cells) or Extracellular Vesicles (Mesenchymal Stem Cells) Transfected or Not With Smad2/3 siRNA

**FIGURE 6:**
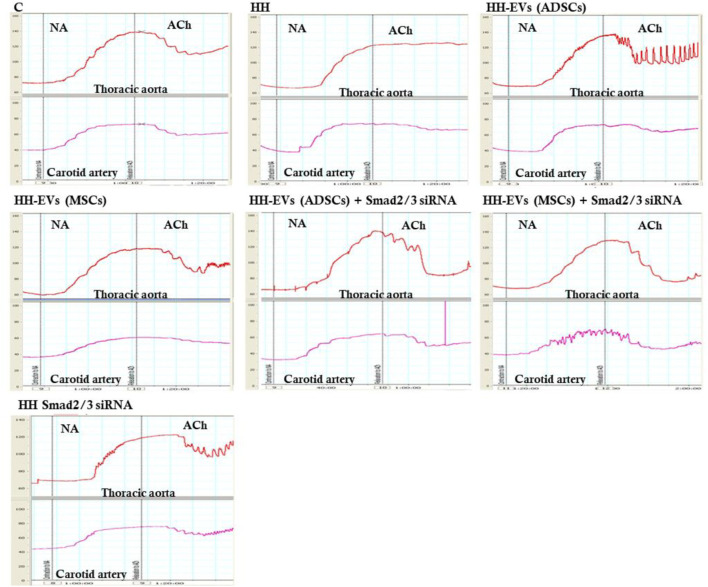
Representative images with myograph recordings at selected time points: for the contraction function to NA (10^−8^ M ÷ 10^−4^ M) and relaxation to ACh (10^−8^ M ÷ 10^−4^ M) in the thoracic aorta (red) and carotid artery (purple) in all investigated experimental groups: C, HH, HH-EVs(ADSCs), HH-EVs(MSCs), HH-EVs(ADSCs)+Smad2/3siRNA, HH-EVs(MSCs)+Smad2/3siRNA, and HH-Smad2/3siRNA. Images were recorded with LabChart 7 software.

**FIGURE 7:**
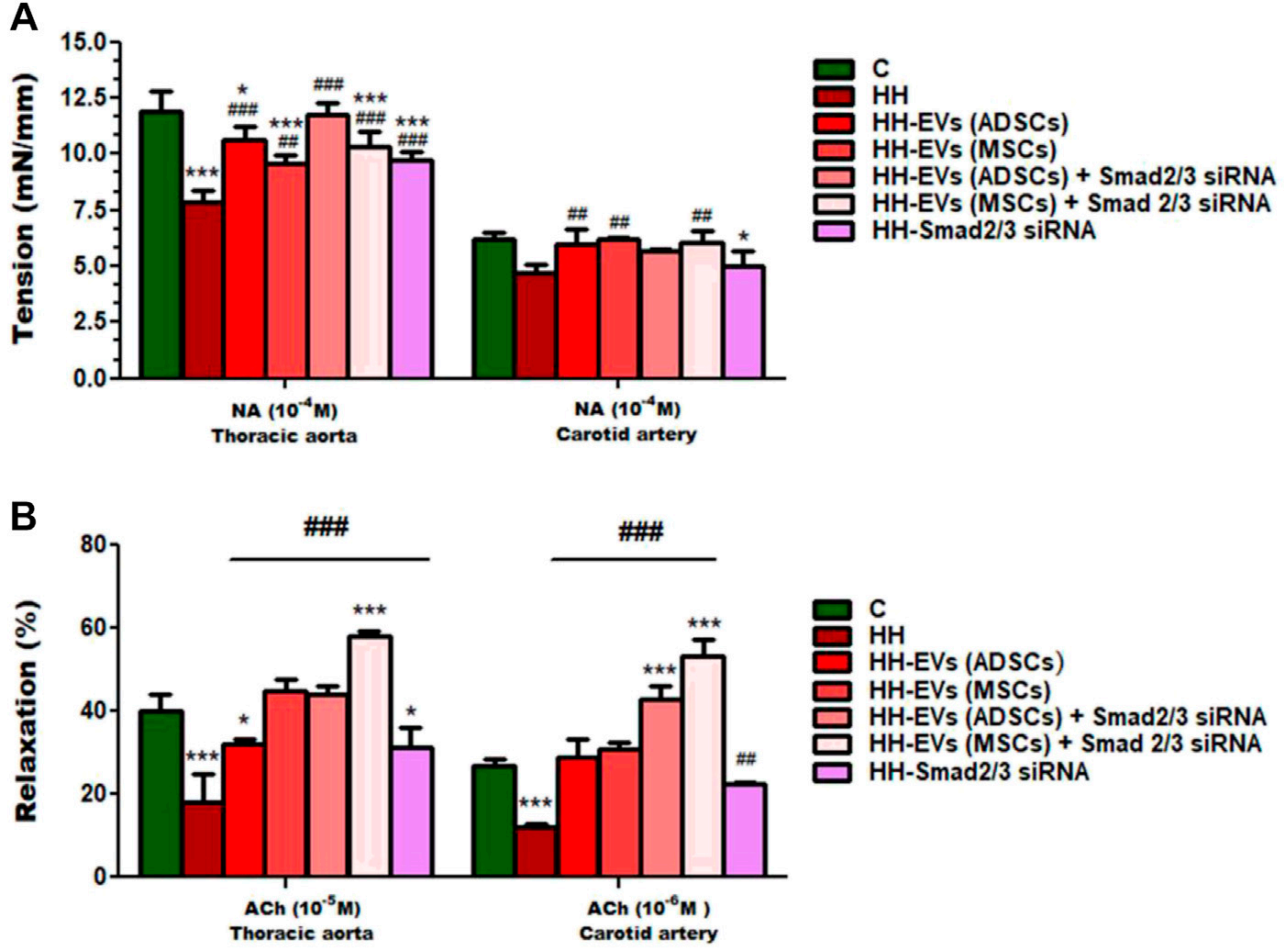
Measures of vascular reactivity of the thoracic aorta (left) and carotid artery (right) explanted from all hamster groups (C, HH, HH-EVs(ADSCs), HH-EVs(MSCs), HH-EVs(ADSCs)+Smad2/3siRNA, HH-EVs(MSCs)+Smad2/3siRNA, and HH-Smad2/3siRNA) by using the myograph technique, in terms of **(A)** contraction to NA and **(B)** relaxation to ACh. Maximal contractile force developed by the thoracic aorta and carotid artery was measured to be 10^−4^ M NA, and maximal relaxation values were recorded to be 10^−5^ M ACh for the thoracic aorta and 10^−6^ M ACh for the carotid artery. Data are mean ± SD of four independent experiments for each investigated treated group and five independent experiments for control and HH groups. The statistical significance, noticeably different, was represented as ***p < 0.005 and *p < 0.05 versus control group and ^###^ *p* < 0.005 and ^##^ *p* < 0.01 versus HH group. The values were calculated by two-way ANOVA and Bonferroni post-test. Enhanced plasma TGF-β1 and AngII levels in atherosclerosis are reduced after the administration of EVs (ADSCs) or EVs (MSCs) transfected or not with Smad2/3 siRNA.

To explore whether the atherogenic diet or treatment administration has the desired effects, namely to induce pathophysiological changes specific to the atherosclerosis process or recover them, changes in vascular tone were analyzed (Figures 6, 7). Using the myograph technique, the ability to contract and relax of the thoracic aorta and carotid artery, in all experimental animal groups, was investigated. It is known that the regulation of vascular tone is controlled by the contractile/relaxing capacity of SMCs, and any change in response can be correlated with the vascular endothelium damage. The experimental results revealed that in both arteries (thoracic aorta and carotid artery), the maximum values recorded at 10^−4^ M NA-induced contraction and 10^−5^M/10^−6^ M ACh-induced relaxation were significantly reduced in the HH group compared with those of the control group (Figures 6, 7), indicating the onset/debut of vascular endothelial dysfunction as a result of atherogenic diet. Isometric force measurements using the wire myograph technique on the thoracic aorta and carotid artery showed that the contractile response was 1.58 times and 1.06 times, respectively, lower in the HH group than that of the C group (Figure 7A). As for the endothelium-dependent responses to ACh, these were 2.10 times reduced in the thoracic aorta and 2.18 times reduced in the carotid artery when comparing the HH group with those of the C group (Figure 7B). Treatment with EVs (ADSCs) alone or in combination with Smad2/3 siRNA re-established the contractile function of the thoracic aorta at values close to normal (Figure 7A). The other types of treatment with EVs (MSCs) alone or in combination with Smad2/3 siRNA and Smad2/3siRNA alone significantly increased NA contractile response of the thoracic aorta compared to that of the HH group but not as good as the one reported for EVs(ADSCs) (Figure 7A). In the case of the carotid artery, no difference in tension developed to the NA vasoconstrictor agent could be distinguished between all groups of treated animals and the values being relatively close to those of the control group, meaning that all types of treatments had a similar contribution to improving contractile function (Figure 7A). Likewise, the treatment with the stem cell-derived EVs alone or in combination with Smad2/3 siRNA was favorable in terms of restoring the ability of both blood vessels investigated to relax, helping to improve the functionality initially affected (Figure 7B). The best ACh relaxation response was observed at the HH-EVs(MSCs)+Smad2/3siRNA group which registered a value above that of the control group (Figure 7B). The arteries from HH-EVs(MSCs) and HH-EVs(ADSCs)+Smad2/3 siRNA animal groups relaxed to very similar values between them, close to those in the control group (Figure 7B). Also, the reported response to ACh of arteries from HH-EVs(ADSCs) and HH- Smad2/3siRNA groups was significantly higher than that from the HH group, but the values were lower than those in the control group (Figure 7B). Figure 6 shows the representative recordings obtained with the help of LabChart 7 software for NA contraction and ACh relaxation responses for each individual experimental group. Contraction to NA (10^–8^ M-10^–4^ M) was measured as the tension developed in the vascular wall (mN/mm), and endothelium-dependent relaxation to ACh (10^–8^ M-10^–4^ M) was calculated as a percentage of the maximum NA pre-contraction. All these data indicate that treatment administered to the HH group not only ameliorates vascular dysfunction but also improves the functionality of the blood vessel. These results reinforce the fact that stem cell-derived EV -based treatment improves vascular wall integrity in terms of structuring the functionality.

**FIGURE 8:**
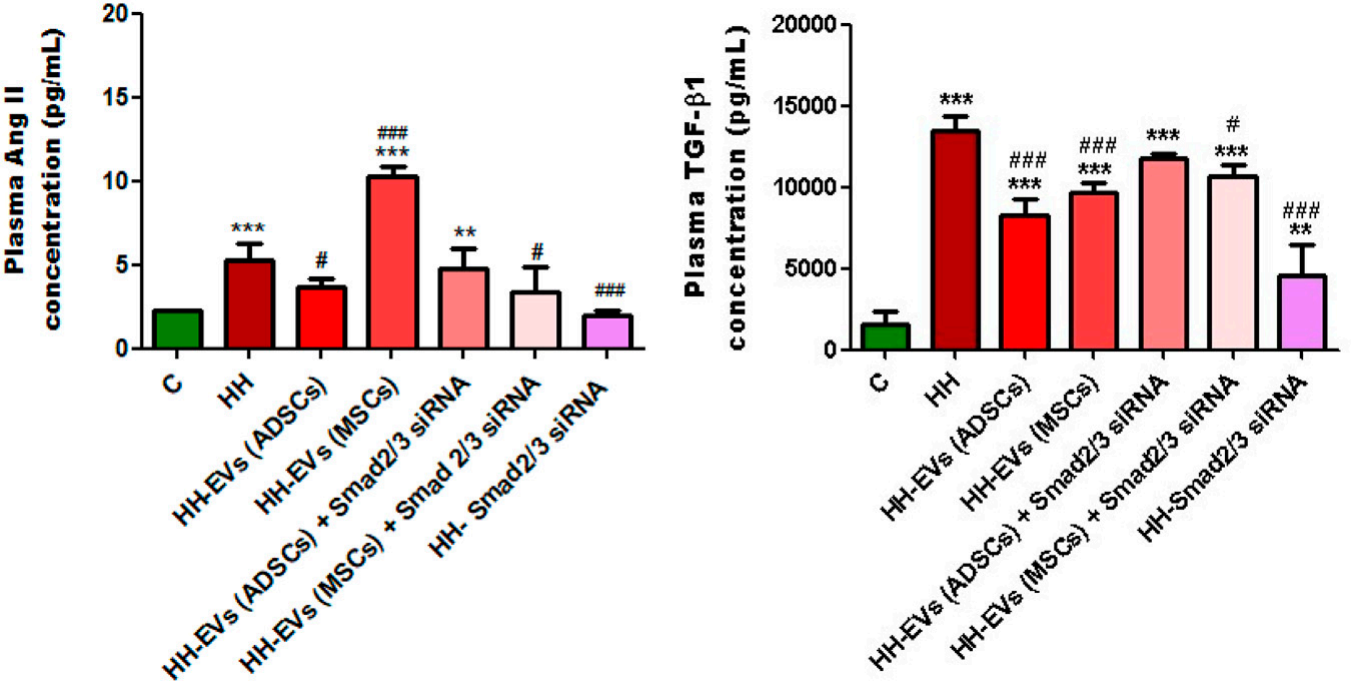
Analysis of plasma TGF-β1 and AngII levels by the enzyme-linked immunosorbent assay (ELISA) method, for all experimental groups: C, HH, HH-EVs(ADSCs), HH-EVs(MSCs), HH-EVs(ADSCs)+Smad2/3siRNA, HH-EVs(MSCs)+Smad2/3siRNA, and HH-Smad2/3siRNA. The measurements were performed in triplicate, and the results were depicted as mean ± SD. The statistical significance, noticeably different, was represented as ***p < 0.005, **p < 0.01 versus control group and ^###^ *p* < 0.005, ^#^ *p* < 0.05 versus HH group. The values were calculated by two-way ANOVA and Bonferroni post-test.

In order to analyze the effects that the administered atherogenic diet has on the morphogenic cytokine profile *in vivo*, we measured the plasma levels of TGF-β1 and AngII obtained by collecting blood from the retro-orbital venous plexus from all groups of experimental animals using the ELISA technique (Figure 8). These two molecules cause the activation of the transcription factors Smad 2/3, NF-kB, and ATF-2, which increase the expression of the inflammatory markers that we analyzed later and whose expression also proved to be elevated. The results showed that the plasma levels of both TGF-β1 and AngII were statistically significantly increased in the HH group compared to those of the control group (***p < 0.005) (Figure 8). Enhanced plasma TGF-β1 and AngII levels correlate with identified structural and functional changes in the thoracic aorta and carotid artery isolated from hamsters in the HH group, which mimic human atherosclerosis.

For groups of hamsters who received treatment, the plasma TGF-β1 and AngII levels were much lower overall, with the exception of the HH-EVs(MSCs) group in which AngII levels were not reduced (Figure 8). Treatment based on EVs from ADSCs or MSCs had a slightly better effect on reducing TGF-β1 levels than treatment based on EVs transfected with Smad2/3 siRNA, but without statistical significance (Figure 8). The biggest reduction in TGF-β1 levels was observed in the Smad2/3siRNA group (^###^ *p* < 0.005) (Figure 8). As for plasma, the AngII levels were significantly reduced for the hamsters in HH-EVs (ADSCs), HH-EVs (ADSCs) + Smad2/3 siRNA, HH-EVs (MSCs) + Smad2/3 siRNA, and HH- Smad2/3 siRNA groups compared to those of the HH hamster group (Figure 8). Again, as in the case of TGF-β1, Smad2/3 siRNA-only treatment greatly reduced plasma AngII levels (Figure 8).

It is important to note that stem cell-derived EV-based treatment regressed the HH-caused alterations in TGF-β1 and AngII pro-inflammatory molecule expressions with a key role in the atherosclerosis-induced vascular dysfunction.

### Extracellular Vesicles (Adipose Tissue Stem Cells) and Extracellular Vesicles (Mesenchymal Stem Cells) Transfected or Not With Smad2/3 siRNA Reduced the Expression of Inflammatory Markers in the Wall of the Thoracic Aorta and Carotid Artery Affected by Experimental Hypertension-Hyperlipidemia

**FIGURE 9:**
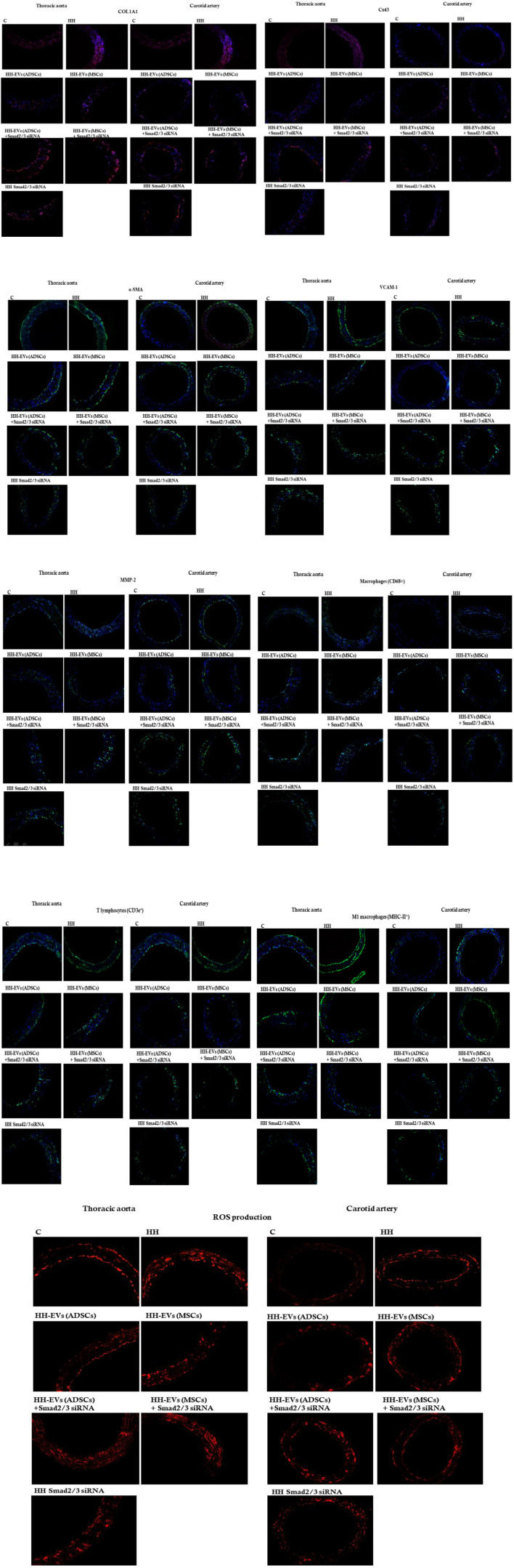
Representative immunofluorescence images for the evaluation of inflammatory markers specific to vascular dysfunction after 4 months of the hyperlipemic–hypertensive diet and the stem cell-derived EV-based treatment or siRNA-based treatment. The thin cryosections from the thoracic aorta (on the left) and carotid artery (on the right) harvested from all experimental groups (C, HH, HH-EVs (ADSCs), HH-EVs (MSCs), HH-EVs(ADSCs)+Smad2/3siRNA, HH-EVs(MSCs)+Smad2/3siRNA, and HH-Smad2/3siRNA) were immuno-labeled for the following: **1)** structural proteins: collagen type I (COL1A1) alpha smooth muscle actin (α-SMA), and connexin 43 (Cx43); **2)** proteins involved in cell adhesion and vascular remodeling: matrix metalloproteinase-2 (MMP-2) and vascular cell adhesion molecule-1 (VCAM-1); **3)** immune cell infiltrate: T cells (CD3e+), total macrophages (CD68^+^), and M1 macrophages (MHC-II+); and **4)** cytosolic ROS production (dihydroethidium (DHE) was oxidized by cytosolic ROS to fluorescent ethidium bromide that intercalates DNA yielding a bright red nuclear fluorescence). Nuclei were shown in blue fluorescence by DAPI dye staining. Each experiment point was performed in triplicate, from two different sets of experiments. A total of five different microscopic fields for each experimental point were analyzed. Total magnification: ×20. The images were quantified using the ImageJ program.

To investigate consequences of atherogenic diet and stem cell-derived EV-based treatment on the vascular wall health, the protein expression of some specific inflammatory markers was analyzed by immunofluorescence performed on sections of the thoracic aorta and carotid artery isolated from all animal experimental groups (Figure 9; Table 2). The examined pro-inflammatory markers as part of the atherosclerotic process were collagen type I (COL1A1) that intervenes in the fibrotic process, connexin 43 (CX43) which forms the myo-endothelial gap junctions, α-SMA which plays an important role in the SMC contraction, VCAM-1 with role in cell adhesion, and MMP-2 with involvement in ECM degradation and vascular remodeling. Also, the total macrophages (CD68^+^), M1 pro-inflammatory macrophages (MHC-II^+^), and the immune cell infiltrate represented by T lymphocytes (CD3e^+^) that support the inflammatory process characteristic of atherosclerosis were followed (Figure 9). Our results showed that all these investigated inflammatory markers had a significantly increased level of protein expression at the HH group compared to that of the C group, meaning that these pro-inflammatory *markers are* abundantly released in the thoracic aorta and carotid artery after 4 months of atherogenic diet (Figure 9; Table 2). The presence of inflammatory markers in the vascular wall may lead to structural and architectural changes recorded by the echocardiography and myograph technique.

**TABLE 2:** Quantification of the stained areas of inflammatory markers from the fluorescence images of the thoracic aorta and carotid artery sections collected from all investigated animal groups. Results were expressed as mean ± SD. The statistical significance, noticeably different, was represented as ***p < 0.005, **p < 0.01, *p < 0.05 values versus control group and ^###^ *p* < 0.005, ^##^ *p* < 0.01, ^#^ *p* < 0.05 values versus HH group. Two-way ANOVA and Bonferroni post-test were applied.

**Stained areas**	**Control (*n* = 11)**	**HH (*n* = 18)**	**HH-EVs (ADSCs) (*n* = 10)**	**HH-EVs (MSCs) (*n* = 9)**	**HH-EVs (ADSCs) +Smad2/3 siRNA (*n* = 5)**		**HH-EVs (MSCs) +Smad2/3 siRNA (*n* = 6)**	**HH-Smad2/3 siRNA (*n* = 8)**
**Alpha smooth muscle actin (α-SMA)**								
Thoracic aorta	160.63 ± 28.9	240.22 ± 19.08 (***p < 0.005)	185.80 ± 25.93 (^###^ *p* < 0.005)	170.17 ± 33.19 (^###^ *p* < 0.005)		149.60 ± 30.13 (^###^ *p* < 0.005)	153.79 ± 15.65 (^###^ *p* < 0.005)	132.16 ± 16.62 (*p < 0.05) (^###^ *p* < 0.005)
Carotid artery	148.75 ± 36.38	236.99 ± 9.46 (***p < 0.005)	202.35 ± 16.78 (**p < 0.01)	180.69 ± 33.51 (^##^ *p* < 0.01)		135.59 ± 24.51 (^###^ *p* < 0.005)	172.04 ± 12.63 (^##^ *p* < 0.01)	143.17 ± 15.94 (^###^ *p* < 0.005)
**Collagen type I (COL1A1)**								
Thoracic aorta	129.25 ± 27.24	248.93 ± 38.96 (***p < 0.005)	114.57 ± 31.04 (^###^ *p* < 0.005)	81.51 ± 22.96 (*p < 0.05) (^###^ *p* < 0.005)		196.17 ± 46.82 (**p < 0.01) (^#^ *p* < 0.05)	225.15 ± 43.90 (***p < 0.005)	181.11 ± 47.84 (**p < 0.01) (^##^ *p* < 0.01)
Carotid artery	217.32 ± 15.49	281.88 ± 12.53 (*p < 0.05)	88.82 ± 23.87 (**p < 0.01) (^###^ *p* < 0.005)	97.98 ± 21.49 (**p < 0.01) (^###^ *p* < 0.005)		221.27 ± 48.36	207.76 ± 63.62 (^##^ *p* < 0.01)	103.77 ± 38.19 (*p < 0.05) (^###^ *p* < 0.005)
**Connexin 43 (Cx43)**								
Thoracic aorta	138.09 ± 12.14	211.94 ± 19.87 (***p < 0.005)	46.34 ± 19.01 (***p < 0.005) (^###^ *p* < 0.005)	118.01 ± 43.88 (^###^ *p* < 0.005)		135.64 ± 17.53 (^###^ *p* < 0.005)	102.72 ± 31.16 (*p < 0.05) (^###^ *p* < 0.005)	47.39 ± 16.05 (***p < 0.005) (^###^ *p* < 0.005)
Carotid artery	46.72 ± 80.4	118.60 ± 34.76 (**p < 0.01)	47.29 ± 19.43	74.79 ± 45.70		98.14 ± 8.02	101.62 ± 18.87	40.69 ± 14.24 (^###^ *p* < 0.005)
**Vascular cell adhesion molecule-1 (VCAM-1)**								
Thoracic aorta	271.15 ± 32.45	489.07 ± 35.31 (***p < 0.005)	152.16 ± 31.72 (***p < 0.005) (^###^ *p* < 0.005)	183.43 ± 44.48 (***p < 0.005) (^###^ *p* < 0.005)		140.59 ± 23.09 (***p < 0.005) (^###^ *p* < 0.005)	173.22 ± 18.97 (***p < 0.005) (^###^ *p* < 0.005)	151.10 ± 15.83 (***p < 0.005) (^###^ *p* < 0.005)
Carotid artery	156.20 ± 26.03	232.08 ± 43.61 (**p < 0.01)	119.19 ± 23.13 (^###^ *p* < 0.005)	119.87 ± 21.03 (^###^ *p* < 0.005)		145.41 ± 20.45 (^###^ *p* < 0.005)	145.94 ± 18.14 (^###^ *p* < 0.005)	132.1 ± 10.36 (^###^ *p* < 0.005)
**Matrix Metalloproteinase-2 (MMP-2)**								
Thoracic aorta	222.97 ± 41.18	460.46 ± 65.94 (***p < 0.005)	88.62 ± 20.53 (***p < 0.005) (^###^ *p* < 0.005)	93.50 ± 25.42 (***p < 0.005) (^###^ *p* < 0.005)		110.45 ± 16.56 (***p < 0.005) (^###^ *p* < 0.005)	109.79 ± 9.10 (***p < 0.005) (^###^ *p* < 0.005)	125.83 ± 27.36 (***p < 0.005) (^###^ *p* < 0.005)
Carotid artery	133.87 ± 15.33	324.68 ± 25.94 (***p < 0.005)	109.00 ± 8.86 (^###^ *p* < 0.005)	91.39 ± 17.20 (*p < 0.05) (^###^ *p* < 0.005)		171.31 ± 15.96 (^###^ *p* < 0.005)	131.99 ± 20.24 (^###^ *p* < 0.005)	102.99 ± 19.66 (^###^ *p* < 0.005)
**T cells (CD3e+)**								
Thoracic aorta	132.10 ± 11.27	231.07 ± 55.07 (***p < 0.005)	112.51 ± 38.95 (^###^ *p* < 0.005)	105.15 ± 30.57 (^###^ *p* < 0.005)		114.76 ± 22.53 (^###^ *p* < 0.005)	98.80 ± 25.07 (^###^ *p* < 0.005)	91.84 ± 19.61 (*p < 0.05) (^###^ *p* < 0.005)
Carotid artery	70.56 ± 11.33	153.98 ± 51.99 (**p < 0.01)	81.18 ± 29.12 (^##^ *p* < 0.01)	117.18 ± 24.66		86.03 ± 11.13 (^#^ *p* < 0.05)	129.89 ± 28.07	96.08 ± 20.76 (^#^ *p* < 0.05)
**Macrophages (CD68** ^**+**^ **)**								
Thoracic aorta	93.50 ± 17.05	246.48 ± 19.18 (***p < 0.005)	100.53 ± 19.18 (^###^ *p* < 0.005)	84.54 ± 25.62 (^###^ *p* < 0.005)		64.45 ± 17.87 (^###^ *p* < 0.005)	75.17 ± 16.55 (^###^ *p* < 0.005)	86.13 ± 19.67 (^###^ *p* < 0.005)
Carotid artery	55.30 ± 3.53	114.67 ± 8.43 (**p < 0.01)	82.31 ± 21.11	129.36 ± 21.03 (***p < 0.005)		39.46 ± 7.01 (^###^ *p* < 0.005)	47.59 ± 22.57 (^###^ *p* < 0.005)	84.97 ± 25.13
**Macrophages M1 (MHC-II+)**								
Thoracic aorta	246.28 ± 20.80	378.20 ± 51.64 (***p < 0.005)	133.00 ± 24.88 (***p < 0.005) (^###^ *p* < 0.005)	206.41 ± 21.71 (*p < 0.05) (^###^ *p* < 0.005)		118.80 ± 15.13 (***p < 0.005) (^###^ *p* < 0.005)	124.06 ± 15.52 (***p < 0.005) (^###^ *p* < 0.005)	131.74 ± 21.32 (***p < 0.005) (^###^ *p* < 0.005)
Carotid artery	71.68 ± 2.63	152.35 ± 17.54 (***p < 0.005)	128.43 ± 24.92 (**p < 0.01)	189.88 ± 45.23 (***p < 0.005) (^#^ *p* < 0.05)		117.25 ± 13.83	119.33 ± 22.83 (*p < 0.05)	104.71 ± 17.49 (^###^ *p* < 0.005)
**ROS (reactive oxygen species)**								
Thoracic aorta	4.76 ± 0.67	7.88 ± 0.88 (***p < 0.005)	4.69 ± 1.16 (^###^ *p* < 0.005)	4.59 ± 0.80 (^###^ *p* < 0.005)		5.61 ± 0.73 (^###^ *p* < 0.005)	5.17 ± 0.83 (^###^ *p* < 0.005)	5.49 ± 0.66 (^###^ *p* < 0.005)
Carotid artery	4.28 ± 0.64	6.58 ± 0.85 (***p < 0.005)	3.82 ± 1.16 (^###^ *p* < 0.005)	4.79 ± 0.88 (^###^ *p* < 0.005)		5.97 ± 0.55 (**p < 0.01)	4.96 ± 1.42 (^##^ *p* < 0.01)	3.85 ± 0.79 (^###^ *p* < 0.005)

Regarding the effect of the treatment administered concomitantly with the atherogenic diet, it was observed that in the case of the inflammatory marker COL1A1 in the thoracic and carotid artery, the treatment with EVs (ADSCs) or EVs (MSCs) generated a strong reduction of its expression, and the calculated values being even lower than those observed in the case of the control group (Figure 9; Table 2). For the other types of treatment applied, the protein expression for COL1A1 had a decreasing trend compared to the values observed in the case of atherogenic diet in both vessels investigated and statistically significant differences being registered in the carotid artery in the case of Smad2/3 siRNA treatment (^###^ *p* < 0.005) (Figure 9; Table 2).

In terms of protein expression for Cx43 and α-SMA, analysis of fluorescence images and their quantification showed that they are significantly higher in the HH group than in the control group in both the thoracic aorta and the carotid artery (***p < 0.005) (Figure 9; Table 2). These results demonstrate that in the structure of the vascular wall, there are changes closely associated with the state of inflammation characteristic of the atherogenic process. Applied treatment based on EVs and Smad2/3 siRNA reduced Cx43 and α-SMA expressions, the best effect being obtained in the case of the HH-EVs(ADSCs)+Smad2/3siRNA and HH-Smad2/3siRNA groups for α-SMA (^###^ *p* < 0.005) and in the case of the HH-EVs(ADSC) and HH-Smad2/3siRNA groups for Cx43, in the thoracic aorta (*p* < 0.005) (Figure 9; Table 2). However, regardless of the group, therapy with EVs transfected or not with Smad2/3 siRNA and the administration of Smad2/3 siRNA as such managed to positively modulate the architecture of the investigated arteries (Figure 9; Table 2). Interestingly, some values were lower than those of the control.

The marker for endothelial activation represented by VCAM-1 had registered values with a fulminant growth trend in the HH group (^***^ *p* < 0.005) compared to those of the control group (Figure 9; Table 2). A considerable improvement observed by lowering VCAM-1 expression levels in both arteries was seen in all groups after treatment administration for 4 months. It should be noted that the treatment made the expression of the protein lower than that measured in control animals (Figure 9; Table 2).

Quantification of MMP-2 expression revealed the same tendency as previously observed in VCAM-1 with an extremely high level in the HH group and with values close to or even lower than those in healthy animals (Figure 9; Table 2). Last, we investigated the inflammatory infiltrate with immune cells (total macrophages, pro-inflammatory macrophages M1, and T lymphocytes) in the arterial wall of the thoracic aorta and carotid artery. The increased averaged values of fluorescence obtained in the HH group showed that the atherogenic diet and gavage significantly enlarged the percentage of infiltration of these cells into the arterial wall compared to that of control animals (Figure 9; Table 2). It could also be seen that after treatment and further administration of the diet, inflammation is reduced, being maintained around control values. The exception was the HH group treated with EVs (MSCs) in which values of fluorescence were equivalent to those from the untreated HH group, indicating that the administration of EVs (MSCs) did not have the desired effect, especially on total and M1 macrophage infiltration in the carotid artery (Figure 9; Table 2).

Analysis of the cytosolic ROS expression level generated in the thoracic aorta of all seven experimental groups revealed that all treated groups had low statistical values compared to those of the HH group (^###^ *p* < 0.005), at which the fluorescence values were significantly increased compared to those measured in group C (^***^ *p* < 0.005) (Figure 9; Table 2). In the case of the carotid artery, the best reduction in ROS levels was observed at the HH-EVs (ADSCs) and HH Smad2/3 siRNA groups (^###^ *p* < 0.005), while the rest of the treated groups also having a significant decrease in the fluorescence level compared to the HH group (Figure 9; Table 2). These results showed that treatment administration greatly diminished ROS release in the vascular wall, thus contributing to the improvement of blood vessel function.

### Extracellular Vesicles (Adipose Tissue Stem Cells) and Extracellular Vesicles (Mesenchymal Stem Cells) Transfected or Not With Smad2/3 siRNA Decreased the Expression Profile of the Key Molecules That Modulate the Inflammatory Response in the Vascular Wall

**FIGURE 10:**
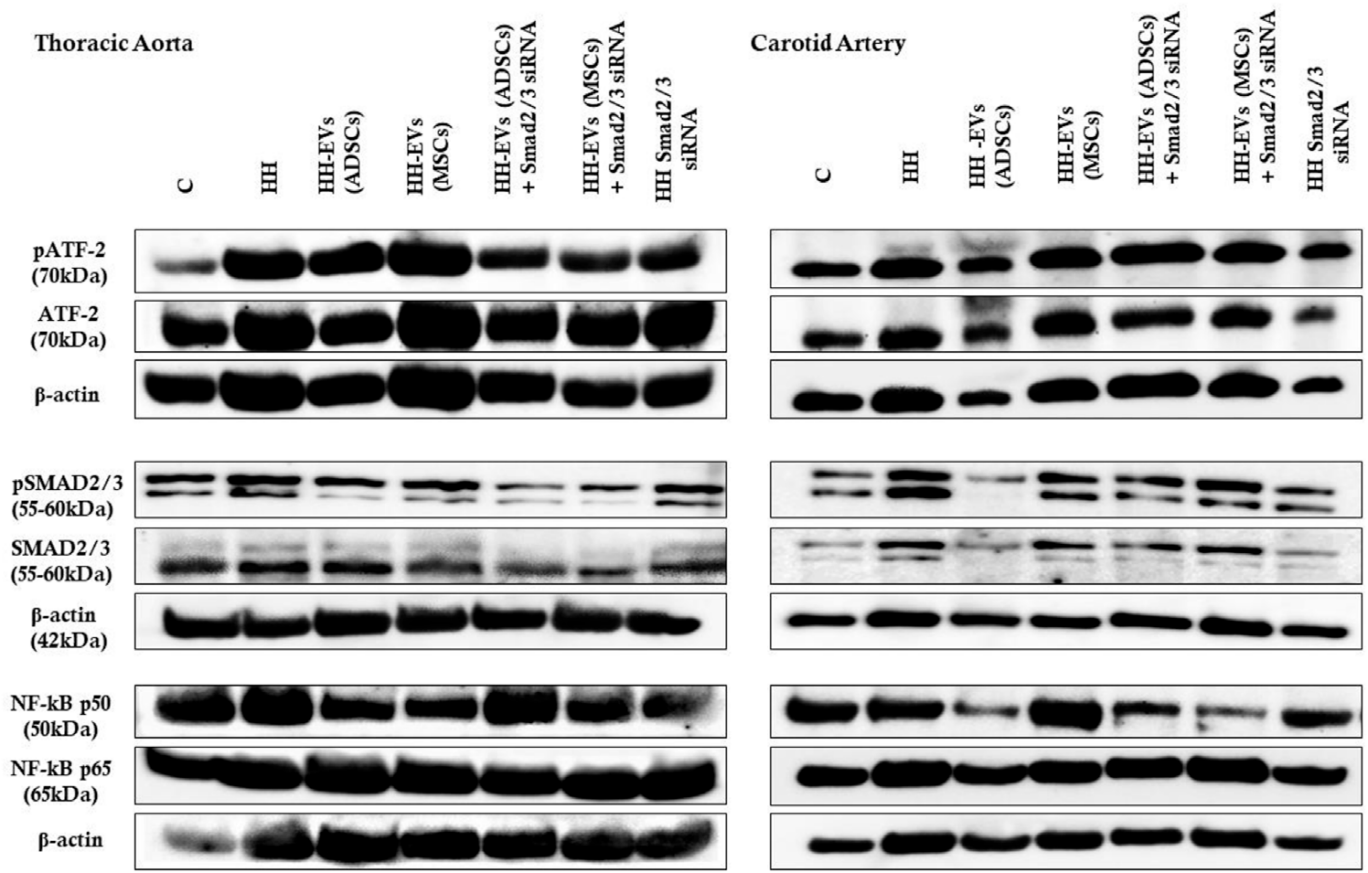
Representative Western blotting images of the expression levels of pATF-2, ATF-2, pSMAD2/3, SMAD2/3, NF-kBp50, NF-kBp65, and β-actin in both thoracic aorta (left) and carotid artery (right) explanted from all seven experimental animal groups.

**FIGURE 11:**
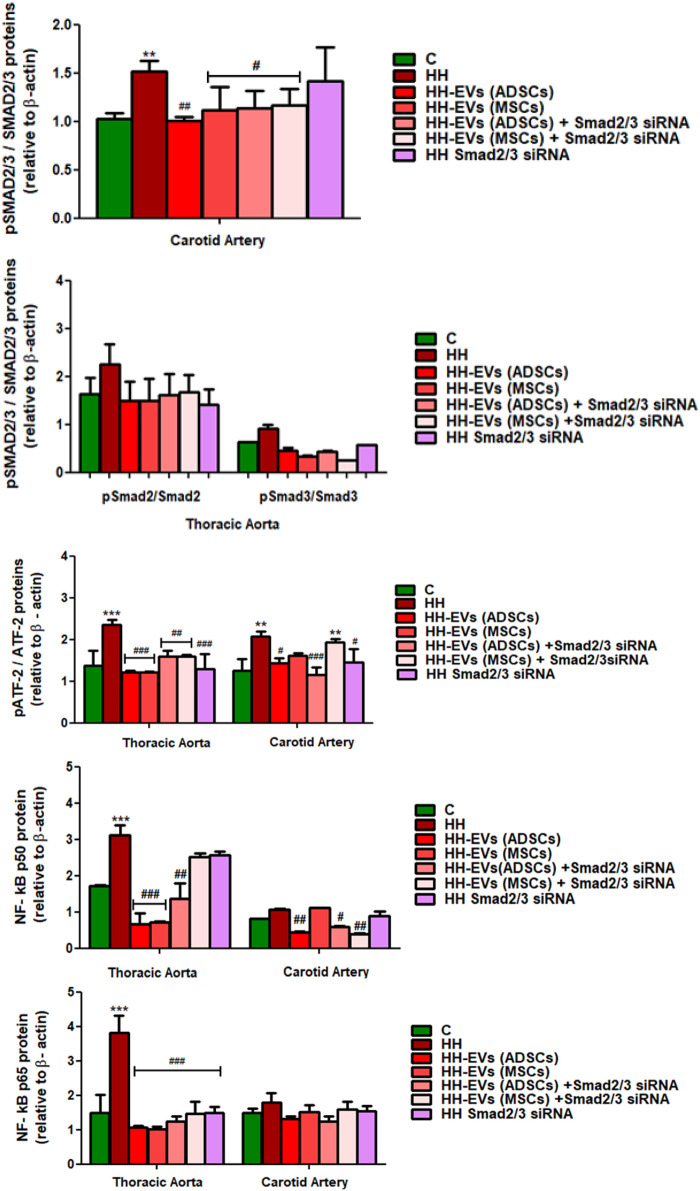
Western Blot analysis for relative expression of specific pro-inflammatory molecules (proteins): pATF-2, ATF-2, pSMAD2/3, SMAD2/3, and NF-kBp50/p65. Histograms show a quantitative representation of the protein levels obtained from all investigated groups of four independent experiments after 4 months of diet and treatment. Each value represents the mean ± SD. The statistical significance, noticeably different, was represented as ***p < 0.005, **p < 0.01 values versus control group and ^###^ *p* < 0.005, ^##^ *p* < 0.01, ^#^ *p* < 0.05 values versus HH group. Statistical analysis was conducted using two-way ANOVA and Bonferroni post-test. The gray intensity of related proteins was analyzed by the TotalLab TL120 program. The housekeeping β-actin protein was used as an internal control for protein normalization and monitor for equal loading. Note that the β-actin expression fluctuated upon the treatment or under physiological and pathological conditions.

**FIGURE 12:**
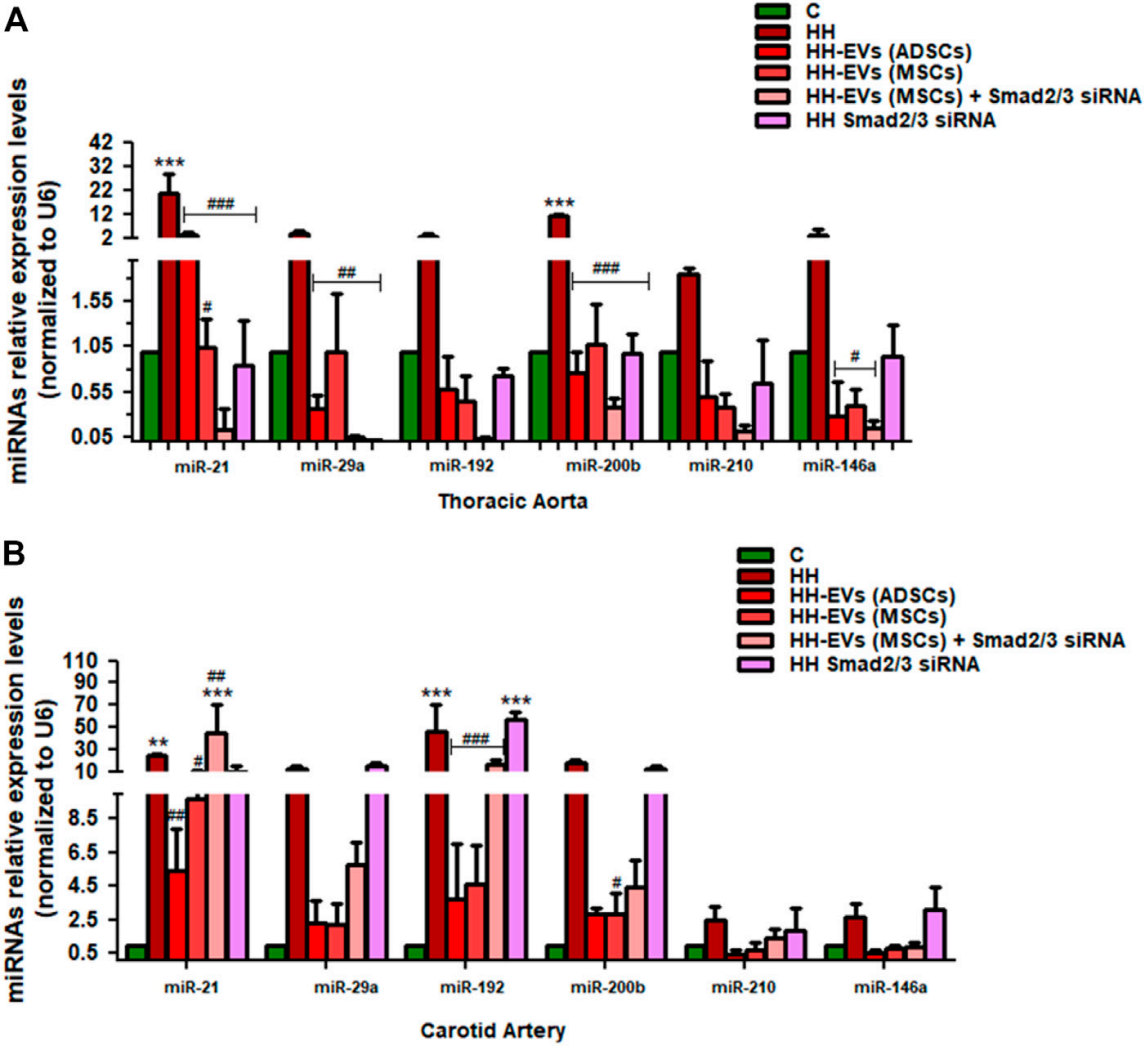
Relative expression levels of six miRNAs (miRNA-21, miRNA-192, miRNA-200b, miRNA-29a, miRNA-210, and miRNA-146a) extracted from two types of tissue **(A)** thoracic aorta **(B)** and carotid artery, explanted from all groups of investigated animals. Total RNA was extracted and used for RT-qPCR. The expression of the miRNA panel was validated using three tissue samples from each artery and matched normal tissue samples. The miRNA expression was normalized using snRU6 as a reference gene. *p*-values of significant differences between the groups were calculated and represented as ***p < 0.005, **p < 0.01, *p < 0.05 for values vs. control group and ^###^ *p* < 0.005, ^##^ *p* < 0.01, ^#^ *p* < 0.05 for values vs. HH group (two-way ANOVA Bonferroni post-test analysis). The mean fold change in expression of the target miRNA was calculated using ∆∆Ct = ΔCt (a target sample) − ΔCt (a reference sample). For the control sample, ∆∆Ct equals 0 and 2° equals 1; therefore, fold change in gene expression relative to control equals 1.

Because our results showed that the plasma levels of AngII and TGF-β1 were increased after atherogenic diet, we thought that the molecules involved in their signaling pathways could be responsible for the changes in structural, functional, and inflammatory markers of the vascular wall in the process of atherosclerosis. As a result, the expressions of transcription factors Smad2/3, ATF-2, and NF-kB p50/p65 (Figures 10, 11) and miR21, miR192, miR200, and miR29 (Figure 12) in the thoracic aorta and carotid artery explanted from all experimental groups were evaluated.

The data showed that the protein expressions of pSmad2/3, Smad2/3, pATF-2, ATF-2, and NF-kB p50/p65 molecules were significantly increased in the thoracic aorta explanted from HH hamsters (***p < 0.005), while in the carotid artery from the HH group only expressions of SMAD2/3 and ATF2 molecules were significantly augmented (**p < 0.01) (Figures 10, 11). These results compared to those in healthy animals claim that the diet accelerated changes at the cellular level with alterations in the regulation of the transcription of numerous genes. The SMAD2/3 transcription factor whose expression was found to be elevated in the HH group was targeted by EV-based treatment by transfecting them with SMAD2/3siRNA. Accordingly, HH animals that were injected with EVs from ADSCs or MSCs transfected or not with Smad2/3 siRNA had a reduced level of SMAD2/3 protein in both types of arteries investigated (Figures 10, 11). No noticeable reduction on SMAD2/3 protein expression was detected following Smad2/3siRNA treatment only, meaning that the presence of EV as a transport system for siRNAs is essential for blocking SMAD2/3.

Regarding the other molecules investigated, the allogenic administration of EVs (ADSCs) or EVs (MSCs) transfected or not with Smad2/3 siRNA and single administration of SMAD2/3siRNA significantly diminished ATF-2 protein expression in the thoracic aorta (^###^ *p* < 0.005, ^##^ *p* < 0.01) and carotid artery (***p < 0.005, **p < 0.01, ^##^ *p* < 0.01) (Figures 10, 11).

Afterward, the expression of NF-kB transcription factor with the two subunits p50 and p65 was followed. For the NF-kB p50 subunit, at the level of the thoracic aorta, the administration of EVs (ADSCs), EVs (MSCs), or EVs(ADSCs) transfected with SMAD2/3siRNA significantly reduced its expression level (^###^ *p* < 0.005, ^##^ *p* < 0.01), while the treatment with EVs(MSCs) transfected with SMAD2/3siRNA or SMAD2/3siRNA generated an insignificant reduction (Figures 10, 11). For the carotid artery, the protein expression of the NF-kB p50 subunit was significantly diminished by EVs (ADSCs) transfected or not with SMAD2/3siRNA and EVs (MSCs) transfected with SMAD2/3siRNA ^(##^ *p* < 0.01, ^#^ *p* < 0.05) (Figures 10, 11).

In parallel experiments, it was shown that the NF-kB p65 expression level was significantly reduced after 4 months of treatment with EVs (ADSCs) or EVs (MSCs) transfected or not with Smad2/3 siRNA or SMAD2/3siRNA in the thoracic aorta, and the values were close to those from the control animals (^###^ *p* < 0.005) (Figures 10, 11). It is worth noting that treatment based on EVs from ADSCs and MSCs had the best results (Figures 10, 11). In addition, NF-kB p65 expression in the carotid artery level was unchanged by all types of treatment administered (Figures 10, 11).

In addition to Smad2/3, ATF-2, and NF-kB p50/p65 molecules, the alterations in the expressions of some miRs could also induce the increased levels of inflammatory markers generating events that contribute to the progression and aggravation of the atherosclerotic lesion. Thus, miRNA-21, miRNA-192, miRNA-200b, miRNA-29a, miRNA-210, and miRNA-146a were selected on the basis of their implication in atherosclerosis, endothelial activation, and inflammation. Each of the selected miRNAs was individually quantified from the thoracic aorta and carotid artery at the end of the experimental period, harvested from the seven experimental groups (Figures 12A,B). The expressions of these miRNAs were compared not only with the HH group but also with the group of healthy animals taken as the control group. The results showed that all selected miRNAs had significantly upregulated levels in the HH hamsters compared to those quantified at hamsters in the control group both in the thoracic aorta and carotid artery (***p < 0.005, **p < 0.01) (Figures 12A,B).

For the entire miRNA panel (miRNA-21, miRNA-192, miRNA-200b, miRNA-29a, miRNA-210, and miRNA-146a) examined in the thoracic aorta, all types of treatment investigated greatly reduced the expression with values in the range of those obtained in healthy animals (^###^ *p* < 0.005, ^##^ *p* < 0.01, ^#^ *p* < 0.05) (Figures 12A).

In the carotid artery, the effect of treatment on the investigated miRNA panel was approximately the same, except for the allogenic administration of EVs (MSCs) transfected with Smad2/3siRNA that had no positive therapeutic effects on miR21, miR-192, miR-210, and miR-146a levels (Figures 12,B), and Smad2/3siRNA alone failed to reduce the expression of the six miRNAs investigated but instead recorded similar values to those of HH animals (Figures 12,B).

It can be concluded that EV (ADSC)-based therapy has a better overall outcome which makes a significant contribution to the suppression of diet-induced changes in the inflammatory process.

## Discussion

It is already known that atherosclerosis is an arterial chronic disease with a progressive evolution in which increased oxidized LDL levels, accumulation of immune cells, and reduced NO bioavailability contribute to the onset of inflammation and subsequently to endothelial damage, severe lesions, and thrombus formation.

In this context, we hypothesized that delivery of extracellular vesicles (EVs), recognized for their potential as therapeutic targets and tools, could restore impaired arterial function in atherosclerosis. Accordingly, we explored the potential beneficial effects of EVs from subcutaneous adipose tissue stem cells (EVs (ADSCs)) or bone marrow mesenchymal stem cells (EVs (MSCs)) transfected or not with Smad2/3 siRNA on vascular dysfunction and its key molecular players.

Thus, the golden Syrian hamster was used to obtain the experimental model of atherosclerosis, also called the hypertensive-hyperlipidemic (HH) hamster. There are a number of arguments that support the use of this animal model to study the complex process of atherosclerosis. It is known that the hamster has been used as an experimental model of induced atherosclerosis since the early 1980s because it has a number of advantages: a low rate of endogenous cholesterol synthesis, secretion of apolipoprotein B-100 by the liver, and complete assimilation of LDL-cholesterol by the receptor involved in the signaling pathway. It has also been shown that the morphology of foam cells formed or lesions in the thoracic aorta are similar to those present in humans (Dillard, Matthan, & Lichtenstein, 2010; Simionescu et al., 1996; Simionescu, Sima, Dobrian, Tirziu, & Simionescu, 1993).

The starting point in characterizing our animal model of atherosclerosis was the evaluation of the basic plasma parameters known to be drastically altered after the administration of the atherogenic diet. The first biochemical changes were observed as early as the second week of the atherogenic diet, so we decided that treatment should be begun at that time. Throughout the experiment, we wanted to investigate the effects of EV-based therapy under conditions of high-fat diet and saline for 4 months and whether it brings improvements in the regression of the atherosclerotic changes in the structure, function, and inflammatory markers of the arterial wall.

The obtained results showed that by comparison with the HH group examined for 4 months on the atherogenic diet enriched with sodium chloride, the administration of EVs(ADSCs) or EVs(MSCs), transfected or not with Smad2/3siRNA, or Smad2/3 siRNA alone of HH animals induced a significant reduction in the plasma levels of total cholesterol, LDL-cholesterol, and triglycerides. Also, the milky white plasma full of lipids, along with the presence of hepatic steatosis in the HH group, was no longer a surprise to us, but it was like a reconfirmation that the diet accelerates the development of atheroma plaque, with rapid progression of atherosclerosis in the arterial sector (Georgescu et al., 2012; Georgescu et al., 2016; Alexandru et al., 2020).

Our findings also validate numerous data being present in the literature that support the fact that this animal model is indicated in the study of vascular pathophysiological changes attributed to atherosclerosis. Thus, we have shown here the presence of the structural and functional changes at the vascular level in the HH group, which are the consequence of disrupting the integrity of vascular endothelium. Our subsequent echocardiographic recordings spread the light on the effect of treatment on the alterations already present in the HH group. The arterial wall thickness observed at the HH group may be associated with the migration of SMCs at the subendothelial level along with the formation of fibro-lipid plaque characteristic of the atherosclerotic process. The inner diameter of the thoracic aorta, although slightly reduced in the case of the HH group, did not significantly show different changes in the control and treated hamsters. Reduced vascular distensibility recorded at the HH group is the result of stiffening of the vascular wall, a process characteristic of atherosclerosis. Thickened vascular wall, reduction of the inner diameter, and stiffening of the wall detected at the HH group by echocardiography may be the consequences of changing the lipid profile, the deposition and accumulation of cholesterol (mainly LDL particles), and fatty substances in the arterial wall and possibly of the formation of atherosclerotic plaque (atheroma) (Pirillo, Bonacina, Norata, & Catapano, 2018; Milutinovic, Suput, & Zorc-Pleskovic, 2020). With the aim of better outlines the functionality of the blood vessel highlighted by echocardiography, we resorted to myographic experiments. Thus, we saw in real time the capacity of contraction and relaxation of the arterial wall for the two blood vessels investigated, namely the thoracic aorta and carotid artery. Following the analysis of the results, we were able to conclude that the animals which received a high-fat diet showed severely impaired functionality compared to control animals, which supports the results from echocardiography. All these results, which show that the wall structure of the thoracic aorta and carotid artery is affected in the case of the HH group, are characteristic of atherosclerotic cardiovascular disease.

Also, from our obtained data, we can appreciate that the administration of EV-based treatment to HH animals, in which the disease has already settled, had beneficial effects on the structure and function of the vascular wall. More specifically, the echocardiographic results of our study revealed that the allogenic administration of EVs (ADSCs) or EVs (MSCs) transfected or not with Smad2/3siRNA or Smad2/3siRNA alone significantly reduced the thickness of the vascular wall of the thoracic aorta/carotid artery, had no significant changes on inner diameter of the arterial wall (thoracic aorta/carotid artery), and significantly diminished values for VTI and velocity of the thoracic aorta compared to those of HH. Note that, EV-based treatment from ADSCs and the one transfected with Smad2/3 siRNA had a better result in restoring the elasticity/distensibility of the wall of the thoracic aorta. Moreover, our results showed a favorable trend in terms of restoring arterial functionality, with values that remained within the scope of those obtained at control. Specifically, the treatment was capable to improve vascular reactivity, respectively, the contractile and relaxing responses of thoracic aorta and carotid artery: the treatment with EVs (ADSCs) transfected or not with Smad2/3 siRNA re-established the contractile function of the investigated vessels to values ​close to normal, while the best response to relaxation was observed in the HH-EVs(MSCs) group transfected with Smad2/3 siRNA which registered a value above that in the control group in both investigated arteries. This fact proves that this pathology accompanied by vascular dysfunction can be remedied/regressed by treatments based on EVs transfected or not with Smad2/3siRNA. All these results regarding structural and functional changes of the vascular wall in atherosclerosis and their regression following treatment with EVs are supported by our previous work in which we only investigated the effect of microvesicles (Alexandru et al., 2020).

The next step in our study was to investigate specific inflammatory markers that could generate vascular dysfunction in our atherosclerosis model and that could be targets of proposed therapy. It is generally agreed that Ang II is a potent vasoconstrictor that activates TGF-β1, a cytokine involved in the regulation of various intracellular signaling cascades, especially of the SMAD-independent pathway. Any disturbance in the signaling pathway could lead to pathophysiological changes in vasculature with an impact on cardiovascular disease.

In this context, the influence of atherogenic diet or of EV-based treatment on plasma TGF-β1 and AngII levels was investigated. The analysis of these parameters at the HH group showed a marked increase in them, meaning that the inflammatory process is strong and sustained. Also, the results exhibited a correlation directly proportional between plasma TGF-β1 and AngII levels and identified structural and functional changes in the thoracic aorta and carotid artery from HH hamsters. For groups of hamsters who received treatment, the plasma TGF-β1 and AngII levels were much lower overall, with the exception of the HH-EVs (MSCs) group in which AngII levels were not reduced. As a result, we could conclude that therapy with EVs transfected or not with Smad2/3 siRNA reduced the inflammatory condition, thus helping to regress the atherosclerosis-associated dysfunction already installed. There are no published data to show the beneficial effect of stem cell-derived EVs on the plasma TGF-β1 and AngII levels in the atherosclerotic process.

In order to have a better overview of the consequences that the activation of key regulatory molecules has on the atherogenic process, we focused on investigating specific structural and inflammatory markers by immunofluorescence. As far as we know from extensive studies, the increased expression of many proteins has been directly associated not only with changes that occur at the time of vascular endothelial damage/injury but also with the complications that follow. In this sense, a first important experimental observation of our study was about the histological structure of the vascular wall, which in the HH group showed disorganization in the architecture of the tunics in the two types of blood vessels examined (thoracic aorta and carotid artery). Also, COL1A expression in the thoracic aorta and carotid artery and Cx43 expression in the thoracic aorta almost increased double at animals with atherogenic diet (HH group). These increases were attributed to the growth of plasma TGF-β1 and AngII levels found by us at the HH group. Our results are consistent with other studies that have shown changes in the vascular wall composition, namely the increase of COL1A1 synthesis due to intensification of the inflammatory process and the activation of the cytokine TGF-β1 which supports the fibrotic process through its response to mechanical stress. Also, the expression of Cx43 was found to be induced in endothelial cells exposed to disturbed flow (Morel, 2014). In addition, our results revealed VCAM-1 and MMP-2 overexpression in endothelial cells, respectively, in SMCs, in the vascular wall of the thoracic aorta and carotid artery after the administration of the atherogenic diet for 4 months. VCAM-1, a protein expressed on the surface of activated endothelial cells, is considered an early manifestation of cholesterol-induced atherosclerosis. It recruits monocytes from the bloodstream, participating in their internalization in the vascular wall, thus generating the release of cytokines and chemokines at the site of injury, culminating in the development of vascular diseases such as atherosclerosis (Mu, Chen, Gong, Zheng, & Xing, 2015). Their expression is modulated not only by Ang II but also by oxidative stress, which we observed in the thoracic aorta and carotid artery from the HH group. Oxidative stress crowns and maintains the state of inflammation at the vascular level, and its elevated values can be associated not only with damage to the endothelium of the arteries and its activation but also with an increase in oxidized LDL in the vascular intima (Davignon & Ganz, 2004; Matsuzawa & Lerman, 2014; Pacurari, Kafoury, Tchounwou, & Ndebele, 2014; Kattoor, Pothineni, Palagiri, & Mehta, 2017; Pirillo et al., 2018). Also, it was demonstrated that the increase in vascular permeability, represented by high levels of VCAM-1, leads to the exacerbation of the levels of inflammatory cells, especially of the circulating monocytes those in the subendothelial space differentiating into macrophages (Wu, Li, Hou, & Chu, 2017; Groh, Keating, Joosten, Netea, & Riksen, 2018; Orekhov & Sobenin, 2019).

In our experiments, the selected markers, CD68^+^, CD3e^+^, and MHC-II^+^ had marked increases that reconfirm that the high-fat diet administered to the HH group aggravated the inflammation, influencing all the other molecules investigated.

Following the interpretation of the results, the data obtained claim that the treatment drastically reduced the vascular inflammatory microenvironment. Thus, for groups of hamsters who received treatment consisting of EVs (ADSCs) or EVs(MSCs) transfected or not with Smad2/3siRNA or Smad2/3siRNA alone, the COL1A1, Cx43, VCAM-1 and MMP2 levels were significantly reduced compared to HH group. Likewise, the treatment reduced the expression of total macrophages (CD68^+^), T cells (CD3e^+^), M1 macrophages (MHC-II^+^) in all treated groups, with the exception of the HH-EVs (MSCs) group in which M1 macrophage infiltration was not affected by treatment. In addition, the treatment administration considerably reduced long-term production of cytosolic ROS in the vascular beds (thoracic aorta/carotid artery) from all treated hamster groups, modulating the activity of the main orchestrators of inflammation-mediated atherosclerotic CVD progression. Noteworthy, the effect of stem cell-derived EV treatment on these molecules has not been shown in any other study. Until now EVs were mostly noticed as biomarkers or communication entities in different physiological ant pathological situations. Their potential as drug delivery systems has been recently noticed and disseminated in different medical applications: in cardiac regeneration (Gasecka et al., 2018), cancer treatment and also in nuclear medicine as radionuclide carriers. A recent opinion-paper on EVs as theranostic agents discussed the benefits of radiolabeled EVs in diagnostic and interventional medicine (Stępień and Rząca, 2021).

Furthermore, we were preoccupied with the examination of the mechanism responsible for generating biochemical, structural, and functional changes in the HH group. As plasma levels of Ang II and TGF-β1 were found to be elevated by the atherogenic diet, we questioned whether the SMAD2/3 signaling pathway is responsible for the exacerbated release of inflammatory markers in the vascular wall. Thus, the next step was to investigate other molecules involved, which in turn transduce signals to the nucleus and control the activity of genes with role in various cellular processes. Consequently, the analysis of protein expression revealed a marked increase in transcription factors Smad2/3, ATF-2, and NF-kBp50/p65 responsible for maintaining the inflammatory state in the arterial wall from the HH group. The increased expression of these molecules is mainly attributed to not only the fat-rich diet administered for 16 weeks but also the way in which the activation of key mediators of inflammation causes the cascade release of other molecules involved, thus maintaining the progression and aggravation of atherosclerotic injury/lesion. For example, NF-kB activation by oxidative stress and inflammation could suppress contractility in SMCs, and in this way it could generate the vascular dysfunction associated with atherosclerosis. As for the effect of therapy on these key molecules, our data exhibited that the allogenic administration of EVs (ADSCs) or EVs (MSCs) transfected or not with Smad2/3 siRNA and single administration of SMAD2/3siRNA reduced their protein expression in the vascular bed. There were some peculiarities that are worth mentioning, namely the treatment with EVs (MSCs) transfected with Smad2/3 siRNA generated an insignificant reduction on the NF-kB p50 expression level, while the NF-kB p65 expression level was unchanged in the carotid artery.

In our study, we were concerned to further examine the mechanism responsible for generating inflammatory markers in the arterial wall with a role in the structural and functional changes observed in the thoracic aorta and carotid artery isolated from the HH group. Consequently, we decided to choose a panel of six miRNAs (hsa-miR-21, hsa-miR-192, hsa-miR-200b, hsa-miR-29a, hsa-miR-210, and hsa-miR-146a) whose expressions are known to be upregulated in the atherosclerotic lesion/fibroatheroma following the release of pro-inflammatory cytokines. Recent studies have shown that there are a multitude of miRNAs associated with the atherogenic process whose expression profiles differ significantly between atherosclerotic plaques and control arteries. In human atherosclerotic lesions, miR-21 expression was observed to be upregulated. This increase was correlated and promoted by the activation of TGF-β1 which determines the posttranscriptional processing of the primary transcript of miR-21 by the Drosha complex. The sheer stress exerted on the endothelial cells induced the overexpression of miR-21 and in VSMCs it determines the change of the phenotype into a synthetic, proliferative one, promoting neointimal growth (Weber, Baker, Moore, & Searles, 2010). Also, miR-146a plays a very important role in promoting the proliferation of VSMCs *in vitro* and vascular neointimal hyperplasia *in vivo* because it targets Krüppel-like factor (KLF4) by reducing its level (Sun et al., 2011). In addition to the action of the TGF-β1/Smad2/3 signaling pathway on the expression of miR-29a which is involved in a number of processes related to vascular abnormalities present in atherosclerosis such as proliferation and migration of VSMCs, epithelial–mesenchymal transition (EMT), and ECM remodeling and angiogenesis, recent studies have also shown a close link between increased miR-29a expression and oxidized LDL levels. These levels have been found increased in plasma in patients with atherosclerosis and are thought to be responsible for the worsening of the disease. Hence, this association shows a good predictive value and can be a possible biomarker for atherosclerosis (Huang et al., 2016). In patients with ischemia caused by heart failure, circulating levels for miR-192 were markedly increased being associated with acute myocardial infarction (Ali Sheikh et al., 2016). Likewise, miR-200b was found to be significantly elevated not only in stroke patients with enhanced plaque (Kim et al., 2015) but also to promote endothelial cell apoptosis under oxidative stress (Zhang, Cheng, Du, Zhang, & Zhang, 2021). The highest levels in miR-210 expression were also observed at the site of more stable carotid plaques and aberrant expression that leads to endothelial cell apoptosis both *in vitro* and *in vivo*, aggravating the progression of atherosclerosis (Li, Yang, Zhang, & Yang, 2017). In our animal model with diet-induced atherosclerosis, obtained data confirmed the previously mentioned literature on miR-21, miR-192, miR-200b, miR-29a, miR-210, and miR-146a expressions in both the thoracic aorta and carotid artery, providing a better understanding of how these molecules regulate atherosclerosis-prone genes. All of these miRs that we investigated in our study, whose gene expressions were found to be elevated at the HH group, are directly associated with main plasma parameters (cholesterol, LDL-cholesterol, and triglycerides) and inflammatory molecules (Ang II and TGF-β1) from the same experimental group. Also, the EV-based treatment brought a significant improvement on the molecules involved in the Ang II/TGF-β1/Smad2/3 signaling pathway, and the results obtained were correlated with the expression levels of miRNA-21, miRNA-192, miRNA-200b, miRNA-29a, miRNA-210, and miRNA-146a. This therapy had a direct impact on the downregulation of genes expressed primarily in endothelial cells, influencing their response to a variety of pathophysiological stimuli. Specifically, the treatment with EVs (ADSCs) or EVs (MSCs) transfected or not with Smad2/3 siRNA or SMAD2/3siRNA alone diminished the expression of miRNA-21, miRNA-192, miRNA-200b, miRNA-29a, miRNA-210, and miRNA-146a in the thoracic aorta and carotid artery with some small exceptions, namely the allogenic administration of EVs (MSCs) transfected with Smad2/3siRNA had no positive therapeutic effects on miR21, miR-192, miR-210, and miR-146a levels in the carotid artery. These positive effects of EVs (ADSCs) or EVs (MSCs) on the proteins and miRNAs found to be modified in the atherosclerotic vascular wall could be attributed to their biological cargo, especially to the miRNAs they contain. In other words, stem cell-derived EVs significantly decreased specific markers of atherosclerosis-induced vascular dysfunction, and these effects could be partly due to their content in miRNAs. These data may provide new alternatives for therapeutic strategies targeting CVD.

### Study Limitations

There are some limitations of our study that we feel responsible for mentioning, are as follows: **1)** many factors can affect the quality and quantity of the EVs produced from the ADSCs/MSCs, such as cellular confluence, early versus later passage of cells, oxygen concentration, cytokines, and serum content of the medium. To avoid some of these problems, in our study, the MSCs (ADSCs or BM-MSCs) at passage five were kept in a serum-free medium for 48 h in order to release EVs (ADSCs) or EVs (MSCs)**; 2)** EVs, collected from the conditioned media of the ADSCs and MSCs, were stored at −80°C until use but not more than 2–3 weeks. However, experiments characterizing them by flow cytometry, electron microscopy, and zeta nanosizer also performed on these EVs showed that their number and structure were not affected by the freezing/thawing procedure; **3)** because the administration of EVs is systemic, there is a possibility that a fairly high percentage can reach the liver. However, our previous studies in which PKH26-labeled MVs administered systemically were quantified under IVIS spectrum equipment which showed that they reach several organs/tissues (liver, lung, kidney, brain, heart, thoracic aorta, and mesenteric resistance arteries), which explains their beneficial action in the vascular wall; **4)** there are some situations in which EV therapy alone has obviously better effects than therapy with EVs which contain siRNA against SMAD2/3. One explanation could be that the mechanisms involved in the complex process of atherosclerosis are multiple, and SMAD2/3 does not always play a key role in all the processes involved; **5)** regarding the clinical applications of EVs, the potential side effects of such therapy should always be considered. It is important to mention that in our present study, we have not observed any possible side effects of systemic delivery of autologous EVs.

## Conclusion

Based on all the results previously mentioned and on some limitations inherent in an experimental model, we can conclude that our study demonstrates that the diet enriched with butter, cholesterol, and gavage with NaCl administered for 4 months generated the animal model with atherosclerotic cardiovascular disease, with vascular dysfunction. Dietary- and gavage-induced changes were evident both in plasma biochemical parameters (altered lipid profile: elevated plasma concentrations of total cholesterol, triglycerides, and LDL-cholesterol) and in inflammatory molecules (increased levels of Ang II and TGF-β1) that were associated with amplified expression of cytosolic ROS production and Smad2/3 molecule. All these changes lead to the progression of alterations in structural and inflammatory molecules in the arterial wall associated with the onset of vascular dysfunction. We also observed that vascular function was affected by low levels of contractile responses to NA and diminished relaxation to ACh together with increased pulse wave velocity and velocity time integral, arterial wall thickness and reduced internal diameter, and distensibility, in both the thoracic aorta and carotid artery in the HH group. These pathophysiological changes observed in the animal model with diet-induced atherosclerosis reproduce quite faithfully the vascular lesions present in the patient with atherosclerotic cardiovascular disease.

In addition, the characterization of EVs, in terms of size and specific markers for exosomes and microparticles, was an extremely important step in our study, performed before obtaining the experimental animal model, to ensure that their isolation is effective. Then, we checked and optimized the transfection rate with Smad2/3 siRNA to use it further as a treatment for the regression of atherosclerotic disease. These steps formed the basis of the therapeutic strategy we implemented in this study. Although the groups of animals that received treatment also received a diet high in fat and saline, the EV therapy was able to regress the arterial changes already installed. Of all the analyzed data, the best results on therapy were seen in the groups in which the treatment was based on EVs not only from ADSCs mainly but also from MSCs. However, there were also situations in which the transfection of EVs with Smad2/3 siRNA had a better effect, amplifying the ability of ADSCs or MSCs to regress endothelial dysfunction or even the administration as such of Smad2/3 siRNA had a better effect.

At the beginning of the study, we aimed to establish the optimal source for the generation of EVs from ADSCs or MSCs with therapeutic potential. Our data strongly suggest that ADSCs seem to be the best option both in terms of obtaining them in culture and using them as an optimal source for EV release with potentially beneficial effects in restoring endothelial dysfunction. Another very important aspect when it comes to treatment is the fact that the therapeutic effect has been maintained throughout the 16 experimental weeks. Based on these observations, it is reasonable to state that EVs(ADSCs) and Smad2/3 siRNA-based therapy may be a feasible therapeutic option and a promising approach for patients with atherosclerotic cardiovascular disease/vascular pathologies.

## Data Availability

The raw data supporting the conclusion of this article will be made available by the authors, without undue reservation.
